# Widespread, long‐term admixture between grey wolves and domestic dogs across Eurasia and its implications for the conservation status of hybrids

**DOI:** 10.1111/eva.12595

**Published:** 2018-03-08

**Authors:** Małgorzata Pilot, Claudia Greco, Bridgett M. vonHoldt, Ettore Randi, Włodzimierz Jędrzejewski, Vadim E. Sidorovich, Maciej K. Konopiński, Elaine A. Ostrander, Robert K. Wayne

**Affiliations:** ^1^ School of Life Sciences University of Lincoln Lincoln UK; ^2^ Department of Environmental Monitoring and Biodiversity Conservation Italian National Institute for Environmental Protection and Research Bologna Italy; ^3^ Department of Ecology and Evolutionary Biology Princeton University Princeton NJ USA; ^4^ Department 18/Section of Environmental Engineering Aalborg University Aalborg Denmark; ^5^ Mammal Research Institute Polish Academy of Sciences Białowieża Poland; ^6^ Institute of Zoology National Academy of Sciences of Belarus Minsk Belarus; ^7^ Institute of Nature Conservation Polish Academy of Sciences Kraków Poland; ^8^ Cancer Genetics and Comparative Genomics Branch National Human Genome Research Institute National Institutes of Health Bethesda MD USA; ^9^ Department of Ecology and Evolutionary Biology University of California Los Angeles CA USA; ^10^ Present address: Instituto Venezolano de Investigaciones Cientificas (IVIC) Centro de Ecologia Caracas Venezuela

**Keywords:** admixed ancestry, domestic dog, gene introgression, grey wolf, hybridisation

## Abstract

Hybridisation between a domesticated species and its wild ancestor is an important conservation problem, especially if it results in the introgression of domestic gene variants into wild species. Nevertheless, the legal status of hybrids remains unregulated, partially because of the limited understanding of the hybridisation process and its consequences. The occurrence of hybridisation between grey wolves and domestic dogs is well documented from different parts of the wolf geographic range, but little is known about the frequency of hybridisation events, their causes and the genetic impact on wolf populations. We analysed 61K SNPs spanning the canid genome in wolves from across Eurasia and North America and compared that data to similar data from dogs to identify signatures of admixture. The haplotype block analysis, which included 38 autosomes and the X chromosome, indicated the presence of individuals of mixed wolf–dog ancestry in most Eurasian wolf populations, but less admixture was present in North American populations. We found evidence for male‐biased introgression of dog alleles into wolf populations, but also identified a first‐generation hybrid resulting from mating between a female dog and a male wolf. We found small blocks of dog ancestry in the genomes of 62% Eurasian wolves studied and melanistic individuals with no signs of recent admixed ancestry, but with a dog‐derived allele at a locus linked to melanism. Consequently, these results suggest that hybridisation has been occurring in different parts of Eurasia on multiple timescales and is not solely a recent phenomenon. Nevertheless, wolf populations have maintained genetic differentiation from dogs, suggesting that hybridisation at a low frequency does not diminish distinctiveness of the wolf gene pool. However, increased hybridisation frequency may be detrimental for wolf populations, stressing the need for genetic monitoring to assess the frequency and distribution of individuals resulting from recent admixture.

## 1 INTRODUCTION

Hybridisation between defined taxonomic entities can be an important conservation problem when it involves an invasive and a native species, or a domesticated subspecies and its wild ancestor (Wayne & Shaffer, 2016). For example, hybridisation with introduced North American ruddy ducks (*Oxyura jamaicensis*) may endanger the genetic integrity of native Eurasian white‐headed ducks (*Oxyura leucocephala*) (Munoz‐Fuentes, Vila, Green, Negro, & Sorenson, 2007), and hybridisation with the domestic cat (*Felis silvestris catus*) is an important conservation threat to the European wildcat (*Felis silvestris silvestris*) (Lecis et al., 2006). Rapid human population growth and the spread of human‐modified habitats can result in a parallel increase in domesticated species and decline of their wild relatives. Such changes in relative densities can increase the frequency of hybridisation, resulting in extensive introgression of derived “domesticated” gene variants into wild populations. Although such introgression is frequently considered maladaptive, it can also provide novel adaptations to a newly occupied or changing environment. For example, admixture between free‐living Soay sheep and a modern sheep (*Ovis aries*) breed resulted in an introgression of a *TYRP1* gene variant associated with light coat colour, which was favoured by natural selection in Soay sheep (Feulner et al., 2013). Another example comes from Alpine ibex (*Capra ibex ibex*), which was shown to acquire one of its two MHC *DRB* alleles from domestic goats (*Capra aegagrus hircus*) (Grossen, Keller, Biebach, & Croll, 2014). Our understanding of the hybridisation process and its consequences is still limited, and improving this knowledge has both theoretical importance (for understanding the role of hybridisation in speciation and adaptation) and practical applications in wildlife conservation and management of feral domestic populations.

The process of domestication is recent in an evolutionary time frame. The oldest domesticated species, the domestic dog *Canis lupus familiaris*, only diverged from the grey wolf *Canis lupus* between 11,000 and 35,000 years ago (Freedman & Wayne, 2017). Because the divergence between domesticated species and their wild relatives is recent, hybridisation between them is particularly frequent as reproductive isolation has not completely developed (Harrison & Larson, 2014; Randi, 2008). The case of wolf–dog hybridisation is particularly interesting due to extensive morphological, ecological and behavioural differences between the two subspecies, which may affect both hybridisation patterns and the fitness of admixed individuals (Anderson et al., 2009; Fan et al., 2016; vonHoldt, Fan, Ortega‐Del Vecchyo, & Wayne, 2017; vonHoldt et al., 2010; Miao, Wang, & Li, 2017). Achieving a better understanding of wolf–dog hybridisation is also important from the perspective of conservation of the grey wolf, which is a keystone species in terrestrial Holarctic ecosystems. This knowledge may also contribute to better control of feral domestic dogs, which can pose a threat to both wildlife and humans (Gompper, 2014).

Domestic dogs coexist with grey wolves across the entire wolf range in the Holarctic. The relationship between the two subspecies is complex and involves resource competition, predation and disease transmission (Lescureux & Linnell, 2014). The two subspecies interbreed in the wild and produce fertile offspring (Leonard, Echegaray, Randi, & Vila, 2014). The context and relative frequency of different types of wolf–dog interactions is not well understood, partially because the ecology of free‐ranging dogs has not been extensively studied (but see Gompper, 2014). Therefore, although the occurrence of wolf–dog hybridisation is well documented (reviewed in Hindrikson et al., 2017), little is known about its underlying ecological mechanisms. It is unknown whether hybridisation has occurred naturally at similar rate since the divergence of wolf and dog lineages, or if it has become more frequent recently as a result of the decline in wolf abundance and the parallel increase in dog numbers.

Occurrence of wolf–dog hybrids and/or back‐crosses has been reported from most European populations, including Italy (Caniglia et al., 2013; Galaverni et al., 2017; Lorenzini, Fanelli, Grifoni, Scholl, & Fico, 2014; Randi & Lucchini, 2002; Randi et al., 2014; Verardi, Lucchini, & Randi, 2006), the Iberian Peninsula (Godinho et al., 2011; Pacheco et al., 2017), North‐Eastern Europe (Latvia and Estonia—Andersone, Lucchini, Randi, & Ozolins, 2002; Hindrikson, Mannil, Ozolins, Krzywinski, & Saarma, 2012), the Balkans (Moura et al., 2014), and the Scandinavian Peninsula (Vilà et al., 2003). There are considerably fewer studies on Asian wolf populations, but recently the occurrence of wolf–dog hybridisation has been reported from the Caucasus (Kopaliani, Shakarashvili, Gurielidze, Qurkhuli, & Tarkhnishvili, 2014; Pilot et al., 2014) and Iran (Aghbolaghi, Rezaei, Scandura, & Kaboli, 2014; Khosravi, Aghbolaghi, Rezaej, Norani, & Kaboli, 2015; Khosravi, Rezaej, & Kaboli, 2013). All these studies focused on relatively small geographic areas, and therefore little is known about geographic variation in the occurrence and frequency of admixed individuals which, if known, could shed a light on factors that favour hybridisation.

The extent of back‐crossing of hybrids into wolf populations is also unknown. Studies based on microsatellite loci failed to reveal large‐scale introgression of dog alleles into European wolf populations, despite the evidence that hybrids can be reintegrated into wolf populations (Andersone et al., 2002; Ciucci, Lucchini, Boitani, & Randi, 2003; Godinho et al., 2011; Lorenzini et al., 2014; Randi & Lucchini, 2002; Vilà et al., 2003). In contrast, genome re‐sequencing data showed that Eurasian wolf genomes may have up to 25% of dog ancestry, and wolf populations with no signs of dog ancestry are rare in Eurasia (Fan et al., 2016).

Effective management of wolf populations that may be affected by hybridisation requires a clear understanding of how hybrids are defined and identified, and how their presence affects population viability. However, the presence of individuals with varying levels of dog ancestry in a population may make the distinction between pure and admixed individuals ambiguous. Individuals resulting from recent hybridisation are difficult to detect based on morphological features (Lorenzini et al., 2014), which may compromise efforts to eliminate them from wolf populations. Moreover, the introgression of dog alleles into wolf populations is not always maladaptive (Anderson et al., 2009; Coulson et al., 2011), and therefore, it is unclear whether elimination of admixed individuals is always the most appropriate conservation strategy.

International, EU, US and national laws on endangered species conservation lack specific legislation on management of hybrids (Allendorf, Leary, Spruell, & Wenburg, 2001; Trouwborst, 2014; Wayne & Shaffer, 2016). Prevention and mitigation of wolf–dog hybridisation may be essential to comply with the Bern Convention on European Wildlife and Natural Habitats and the EU Habitats Directive. On the other hand, the prohibitions on the killing and capturing of the wolves introduced by these both legal frameworks also cover wild wolf–dog hybrids (Trouwborst, 2014). Therefore, better knowledge of the hybridisation process is needed to guide conservation legislation and practice (Wayne & Shaffer, 2016). In this study, we used genome‐wide SNP data to analyse ancestry in Eurasian grey wolves in order to detect first‐generation wolf‐dog hybrids, recent back‐crosses, and signatures of more distant hybridisation events. This approach allowed us to assess the effect of hybridisation on grey wolves at a continental scale.

## 2 MATERIALS AND METHODS

### 2.1 Data set

This study utilised previously reported genomewide SNP data from wild canids and domestic dogs genotyped on an Affymetrix Canine SNP Genome Mapping Array at 60,584 high‐quality autosomal SNP loci and 851 X chromosome SNP loci (vonHoldt et al., 2010). The original data set of 225 grey wolves, 60 coyotes and 912 domestic dogs (vonHoldt et al., 2010), was previously used in studies focused on dog domestication (vonHoldt et al., 2010), the genetic architecture of morphological traits in the domestic dog (Boyko et al., 2010), wolf–coyote hybridisation in North America (vonHoldt et al., 2011) and signatures of selection in North American wolves (Schweizer et al., 2016). Here, we utilise this data set in a novel way to study wolf–dog hybridisation in the wild.

**Figure 1:**
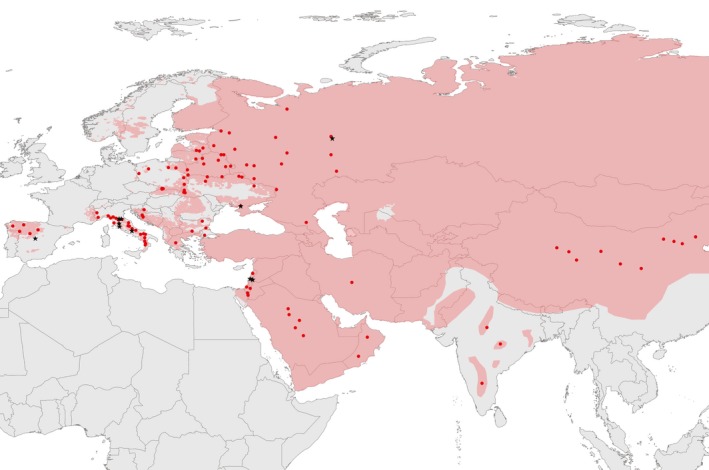
Geographic distribution of the Eurasian wolf samples analysed (red dots), and the F1 hybrid and F2/F3 back‐crosses (black stars) identified in the study. Sample locations in Europe, including Russia, are precise; sample locations in Asia are approximate. The area highlighted in pink represents the wolf distribution range in Eurasia

From the original data set, we selected 252 individuals: 54 Eastern European wolves, 20 Italian wolves, six Iberian wolves, 17 putative wolf–dog hybrids (nine from Eastern Europe and eight from Italy), 28 Asian wolves (five from Saudi Arabia, seven from Israel, two from Oman, one from Iran, three from India, and 10 from China), 125 dogs of different breeds (1–2 individuals per breed), and two free‐ranging nonbreed dogs. Geographic distribution of the samples of wolves and admixed canids is presented in Figure 1. The data set of pure‐breed dogs besides modern breeds of European origin also included ancient breeds. The group of ancient breeds encompasses non‐European breeds, largely of Asian origin, that are genetically distinct from breeds of European origin, as first proposed by Parker et al.(2004). In addition, we included 35 coyote (*Canis latrans*) genotypes from vonHoldt et al. (2010) data set, representing most of the species range (from California to Vermont, and from Alabama to Manitoba). Coyote distribution is limited to North America and therefore they do not interbreed with Eurasian wolves.

Putative wolf–dog hybrids were identified a priori based on genetic analyses using microsatellite loci (Randi & Lucchini, 2002) and/or morphological anomalies as compared to the typical grey wolf phenotype (e.g., dewclaws; Ciucci et al., 2003). Morphological anomalies alone do not allow for reliable identification of wolf–dog hybrids, and therefore we used them for indicative purposes only. We also analysed two individuals from Italy with black coat colouration, which is a dog‐derived trait, but does not necessarily imply a recent admixed ancestry (Anderson et al., 2009; Caniglia et al., 2013; Galaverni et al., 2017). The inclusion of the pre‐identified putative hybrids into our data set allowed us to assess the accuracy of hybrid detection based on a small number of microsatellite loci, but it prevented us from estimating the frequency of hybridisation in the populations studied.

For comparative purposes, we also assessed the occurrence of dog admixture in North American wolves. For this analysis, we used genotypes of 48 individuals from vonHoldt et al. (2010) data set, representing the following populations: Mexico (five individuals), Yellowstone (18), Northern Quebec (six), forest (11), taiga (four) and tundra (three) habitats in Northern Canada, and Vancouver Island, British Columbia (one individual). We excluded individuals from the wolf–coyote hybridisation zone in the Great Lakes Area (vonHoldt et al., 2011). For these populations, we would have to consider dog–wolf–coyote admixture and the outcome of such analysis would not be directly comparable to the analysis of dog–wolf admixture in Eurasia.

### 2.2 Detection of admixed individuals based on global ancestry estimates

To identify signatures of dog ancestry in Eurasian wolf populations and assess the accuracy of prior hybrid identification, we used the Bayesian clustering approach implemented in structure (Pritchard, Stephens, & Donnelly, 2000) and admixture (Alexander, Novembre, & Lange, 2009). We used both structure and admixture, as this allowed us to ensure that the inferred admixture patterns are consistent and thus reliable. This analysis was based on the data set consisting of European wolves, putative wolf–dog hybrids, dogs and coyotes.

Prior to the analysis of population structure, we used PLINK (Purcell et al., 2007) to prune the data set removing SNPs with genomewide pairwise genotypic association coefficient *r* ^2^ ≥ 0.5. Pruning was carried out with 50 SNP sliding windows, shifted and recalculated every 10 SNPs, yielding a set of loci that are not in strong linkage disequilibrium (LD). We also removed loci that were invariant in the analysed sample set, or had minor allele frequency (MAF) below 0.01, resulting in a data set of 53,248 SNPs.

We ran structure using 100,000 MCMC iterations preceded by 20,000 burn‐in iterations with five replicates for K (the number of groups) from 1 to 10 on the pruned data set. We used the correlated allele frequencies and admixture model and checked whether the run parameters reach convergence within the burn‐in period for each K. We used Structure Harvester (Earl & vonHoldt, 2012) to assess the optimal K value, based on likelihood values and Evanno, Regnaut, and Goudet (2005) delta K values.

We ran admixture analysis for K from 1 to 15, using the default termination criterion, which stops iterations when the log‐likelihood increases by <ε = 10^−4^ between iterations. The K value for which the model has best predictive accuracy was identified using a cross‐validation procedure, where the runs are performed after removing 10% of the genotypes at random, with 10 repetitions. We assessed the optimal K as the value that resulted in the lowest cross‐validation error.

To visualise the dominant components of variability in the data set and the position of the putative hybrids relative to wolves and dogs, we carried out the principal component analysis (PCA), using smartpca package from the software eigensoft (Price et al., 2006).

### 2.3 Ancestry block analysis in Eurasian wolves using lamp software

We used lamp (Sankararaman, Sridhar, Kimmel, & Halperin, 2008) to carry out the ancestry block analysis, which infers blocks of wolf and dog ancestry along chromosomes in each individual. This analysis allowed us to assess the admixture status of the putative wolf–dog hybrids, identify additional admixed individuals and assess the signatures of past admixture between wolves and dogs. lamp's unique feature among software performing the ancestry block analysis is that it allows ancestry blocks estimation without defining a priori ancestral populations (wolves and dogs without signatures of past admixture in their ancestry). We used this feature in our analysis, because we were not able to identify a priori which individuals were pure wolves without past dog admixture. Instead, the identification of ancestral populations was an integrated part of the lamp analysis. This was achieved in a similar way to the structure analysis with K = 2, which divides a data set analysed into two genetic clusters without any prior information about population subdivision.

For the lamp analysis we used the data set consisting of wolves, putative hybrids, and pure‐breed dogs. We assumed a mixture proportion of 0.50:0.50, which was the frequency of wolves and putative hybrids (125 individuals) versus dogs (125 individuals). The use of this ratio was based on a conservative assumption that the putative hybrids group with wolves rather than dogs. This assumption is supported by the fact that the set of the putative hybrids can include back‐crosses besides F1 hybrids. Back‐crossing into wolf populations is more likely than into dog populations, especially given that our data set consisted of pure‐breed dogs with breeding patterns controlled by humans.

All SNPs (61K) were included in the initial data set, which was subsequently pruned for loci that were monomorphic for the analysed set of individuals, and for *r* ^2^ > 0.1. We used a recombination rate of 5e−10, and fraction of overlap between adjacent windows (offset) of 0.2. We assumed a recent admixture (10 generations since admixture), because otherwise the power to detect F1 hybrids and recent back‐crosses was diminished: the assumption of 100 generations as admixture resulted in all individuals (wolves and dogs) being admixed.

### 2.4 Ancestry block analysis in North American wolves using lamp software

To assess the signatures of past wolf–dog admixture in North America, we carried out the lamp for North American grey wolves as described for Eurasian wolves. In this case we used 48 pure‐breed dogs (1 individual per breed) to match with 48 wolves, in order to maintain the 0.50:0.50 mixture proportion. All other parameters were the same as described above.

### 2.5 Ancestry block analysis in Eurasian wolves using PCadmix software

To assess the accuracy of the local ancestry inference in lamp, we replicated the ancestry block analysis using PCadmix software (Brisbin et al., 2012). This software does not carry out an unsupervised ancestry assignment, that is it requires prior information regarding allele frequencies in nonadmixed populations. However, it has been shown to have better accuracy than the lamp analysis using a supervised ancestry assignment mode (Brisbin et al., 2012). PCadmix uses an algorithm based on the Principal Component Analysis to determine local ancestry along each chromosome for phased SNP genotype data. We phased the genotypes using fastPHASE (Scheet & Stephens, 2006); the wolf and dog genotypes were phased together, with population information (wolf or dog) provided in an input file. Individuals previously identified as back‐crosses were assigned to the wolf population for the purpose of phasing.

In the comparison between two methods of supervised ancestry assignment, the same set of nonadmixed individuals from ancestral populations would be used. In lamp, the ancestral populations were only identified during the analysis, and therefore, we used the results from lamp to predefine the set of nonadmixed individuals to be used as an input for PCadmix analysis. For wolves, the criteria for individuals to be included to a nonadmixed set were (i) an average proportion of autosomal SNP alleles of dog ancestry, as identified in lamp, lower than 0.005; and (ii) no more than one chromosome having over 10% of SNP alleles of dog ancestry. These criteria were met by 48 wolves. For dogs, we applied more strict criteria, as their overall level of admixture was lower than in wolves. The dogs included in the nonadmixed set had (i) an average proportion of autosomal SNP alleles of wolf ancestry lower than 0.003; and (ii) each chromosome having less than 10% of SNP alleles of wolf ancestry. These criteria were met by 107 dogs. The admixture status of the remaining individuals was assessed using PCadmix. The phased genotypes were pruned from loci in strong linkage disequilibrium (with *r* ^2^ > 0.8), and the analysis was carried out in windows of 20 SNPs. In contrast to lamp, which assumed the markers to be nearly independent and therefore required heavy pruning to achieve *r* ^2^ < 0.1, PCadmix accommodates nonindependent markers and therefore can use more relaxed criteria for LD pruning (Brisbin et al., 2012).

### 2.6 Analysis of X chromosome data

The X chromosome data were analysed for males and females separately. For males, we excluded SNPs from the Pseudo‐Autosomal Region (PAR; first 6 Mb of the X chromosome). Outside the PAR, we found four loci for which 10 or more of 102 males had heterozygous calls. Because we could not explain this observation, we removed these loci from both male and female data sets. At the remaining 508 SNP loci no more than 5 of 102 males displayed heterozygous calls. These were most likely genotyping errors, and were treated as missing data. Although this implies a 5% error rate at some loci, most loci did not display any heterozygous calls, and the overall error rate was 0.075%. This is consistent with the genotyping error rate for the entire microarray data, which was estimated for samples run in duplicates at less than 0.1% (Boyko et al., 2010). After the adjustments described above, we obtained X chromosome haplotypes for the males. We calculated genetic distances between these haplotypes as the proportion of SNP sites at which two haplotypes being compared are different, and constructed the neighbour‐joining tree in MEGA (Tamura et al., 2011). The same set of 508 SNPs was analysed in lamp.

For females, we used the same set of 508 loci as for males to phase the genotypes in fastPHASE, using the homologous male haplotypes as additional input to enhance the results. We constructed the neighbour‐joining tree for the inferred female haplotypes. We also used the entire set of 851 X chromosome SNPs for ancestry blocks analysis in lamp and for population structure analysis in admixture, carried out as described above for autosomes.

### 2.7 Assessment of heterogeneity of dog ancestry proportions across the chromosomes in Eurasian wolves

In recently admixed populations, differences in ancestry proportions may arise among chromosomes as a result of nonrandom mating and selection. We applied the Chromosomal Ancestry Differences (CAnD) test (McHugh, Brown, & Thornton, 2016) to assess whether there are significant differences in dog ancestry contributions among the chromosomes in Eurasian wolves. Details of this analysis are described in Supporting Information, Part A.

### 2.8 Analysis of a data set of European wolves and European dog breeds

We carried out the analyses described above for a data set consisting of European wolves and European dog breeds only, to assess whether the methods we applied provide consistent identification of admixed individuals, which is independent of the composition of the included wolf and dog data sets. Details are described in the Supporting Information, Part B.

### 2.9 Estimation of heterozygosity, autozygosity and linkage disequilibrium

We calculated observed and expected heterozygosity in wolf populations from different parts of Eurasia based on the 61K SNP set for autosomal chromosomes. To minimise the bias in heterozygosity estimates due to sample size, we included only the local populations with at least five individuals sampled and selected a random subset of six individuals from each population where the total sample size was larger. We considered nonadmixed Italian wolves and Italian admixed canids (the admixture status being confirmed/identified in this study) as separate groups. Admixed individuals were also excluded from calculations for other populations, except for Israel, where all individuals carried signatures of past admixture (see Results).

To assess the autozygosity level in admixed individuals and nonadmixed wolves from different regions we identified runs of homozygosity (ROHs) in individual canids spanning at least 25 SNPs and longer than 100 kb. This analysis was carried out using the SNP set pruned for local LD (by removing SNPs with *r* ^2^ > 0.5) to minimise the detection of ROHs that result from strong LD and do not represent autozygosity.

To compare LD levels between admixed and nonadmixed populations we calculated *r* ^2^ between all pairs of autosomal SNPs with a minor allele frequency >0.15 in each European population, based on 5–6 individuals each to minimise sample size effect. We estimated the distance at which *r* ^2^ coefficient decays below 0.5. All the above analyses were carried out in PLINK.

## 3 RESULTS

### 3.1 Identification of admixed individuals using Bayesian clustering methods

**Figure 2:**
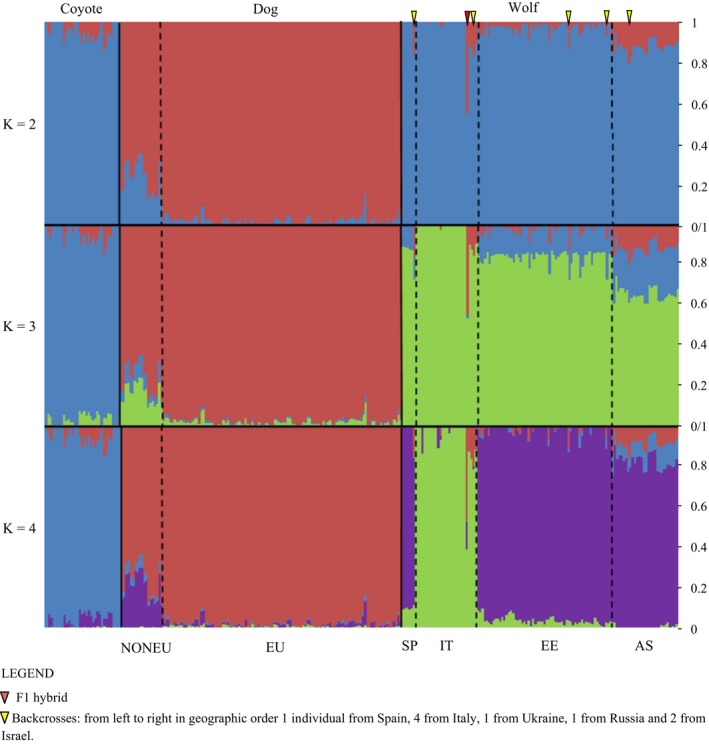
Genetic differentiation between regional populations of wolves and domestic dogs inferred using the program admixture, assuming two, three and four genetic clusters (K). Plots for a broader range of K values are shown in Figure S1. Coyote—*Canis latrans*; dog—*Canis lupus familiaris*: European breeds, non‐European breeds; Wolf—*Canis lupus*: from left to right Spain, Italy, Eastern Europe (Bulgaria, Croatia, Greece, Turkey—European part, Slovakia, Poland, Lithuania, Belarus, Ukraine, Russia), Asia (Israel, Arabia, Oman, Iran, India, China)

Results from structure and admixture were highly consistent for K values between 2 and 4. At K = 2 dogs were distinguished from wild canids, and at K = 3 dogs, wolves and coyotes were identified as the three distinct groups. Italian wolves were indicated as the fourth group at K = 4 (Figure 2, Figure S1). structure and admixture results were inconsistent regarding the optimum number of genetic clusters (K value). Using structure, the highest delta K value was for K = 2 and the second highest for K = 3. In admixture, the lowest cross‐validation error was obtained for K = 6. Both in structure and admixture, the genetic clusters identified at K = 6 included coyotes, two groups of dogs (see below), and clusters of Italian wolves, other European wolves and Saudi Arabian wolves, with other Asian wolves having intermediate assignment values between the European and the Saudi Arabian clusters (see Supporting Information, Figure S1). The clustering patterns in dogs at K = 6 differed between structure and admixture, but these patterns do not affect our inference regarding wolf–dog admixture. The allele frequency divergences among populations estimated in structure are reported in Table S1).

**Figure 3:**
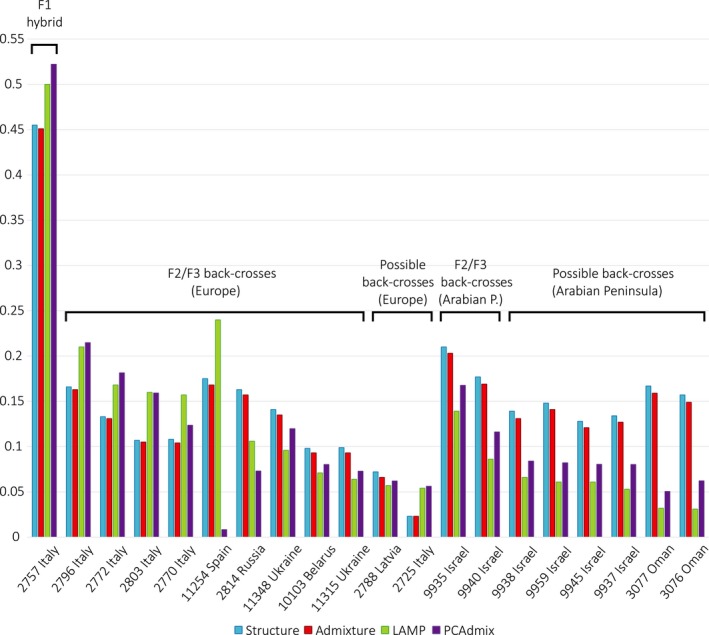
Dog ancestry proportions in the F1 hybrid, F2/F3 back‐crosses and putative further generation back‐crosses, estimated from the ancestry blocks analyses in lamp and PCA dmix, and the analysis of population genetic structure in structure and admixture. Individuals’ admixture status was inferred based on the results from lamp, structure and admixture, while the PCA dmix was carried out as a follow‐up analysis. The lamp and PCA dmix results are presented as the mean percentage of SNP alleles of dog ancestry in autosomal chromosomes. The structure and admixture results are presented as the assignment probability of an individual to the dog cluster (assuming K = 2). The ancestry proportions were calculated for autosomal chromosome data

Clusters identified at K = 2 and K = 3 corresponded to the three canid species/subspecies analysed, which allowed for identification of hybrids and back‐crosses. Among individuals identified a priori as putative hybrids (nine from Eastern Europe and eight from Italy), we identified only one F1 hybrid, Italian canid #2757. This individual had about 45% assignment to the dog cluster in both structure and admixture at K = 2. Four other putatively admixed canids from Italy and one from Eastern Europe had assignment probabilities the dog cluster of 10‐17% (Figure 3, Table S2), suggesting that they were F2 or F3 back‐crosses (offspring of F1 hybrids or F2 back‐crosses breeding with pure wolves). The remaining individuals identified a priori as putative hybrids had assignment probabilities to the dog cluster within the range for nonadmixed wolves.

Two canids from European populations that were assumed a priori to be nonadmixed wolves, had assignment probabilities to the wolf population ~83% and ~86%, respectively, which was outside the range for other European wolves (92–100%), but within the range for F2/F3 back‐crosses (83–90%). Two other individuals from Eastern Europe had ambiguous admixture status, with assignment probabilities to the wolf population of about 90% inferred in both structure and admixture (Table S2).

Asian wolves had higher assignment probabilities to the dog cluster as compared with European wolves, ranging from 11 to 13% in Arabian Peninsula wolves, 9–15% in Chinese wolves, and 6–9% in Indian and Iranian wolves (Table 1). Four canids from the Arabian Peninsula (Israel and Oman) that were assumed a priori to be pure wolves, had assignment probabilities to the dog cluster of 15–21% (Figure 3; Table S2). This was outside the range for other Arabian Peninsula wolves, but within the range for European F2/F3 back‐crosses, so these individuals could be back‐crosses as well. However, both structure and admixture inferred some level of dog admixture in all Arabian Peninsula wolves, and therefore, this inference is less robust compared with that for European canids.

**Table 1:** Summary of the results of the ancestry blocks analyses in lamp for Eurasian wolf populations, North American wolves populations and pure‐bred dogs, in comparison with the results of tests based on the analysis of population genetic structure (using structure and admixture, assuming K = 2)

Canid group	LAMP results				structure	admixture
	Autosomes	No. admixed autosomes	chr X females	chr X males		
Chinese wolves (10)	0.981–1.000	0–3	0.753–1.000	1.000	0.853–0.900	0.861–0.908
Indian and Iranian wolves (4)	0.959–0.996	0	1.000	1.000	0.913–0.930	0.922–0.938
Arabian Peninsula wolves (6)	0.999–1.000	0–6	1.000	1.000	0.866–0.886	0.875–0.893
Arabian Peninsula F2/F3 back‐crosses (2)	0.861–0.914	12–16	1.000	1.000	0.790–0.823	0.797–0.831
Arabian Peninsula F4+ back‐crosses (uncertain)	0.934–0.968	3–10	1.000	1.000	0.833–0.872	0.841–0.879
European wolves (85)	0.949–1.000	0–8	0.964–1.000	1.000	0.922–1.000	0.930–1.000
European F2/F3 back‐crosses (7)	0.760–0.904	12–23	0.877–1.000	1.000	0.825–0.893	0.832–0.896
European F4+ back‐crosses (uncertain) (4)	0.929–0.946	8–11	–	0.849–1.000	0.901–0.977	0.907–0.977
North American wolves (42)	0.994–1.000	0–1	–	–	–	–
Mexican wolves (5)	0.949–0.993	1–7	–	–	–	–
European dog breeds (105)	0.000–0.003	37–38	0.000–0.018	0.000	0.001–0.083	0.000–0.091
Non‐European dog breeds (20)	0.002–0.112	20–38	0.000–0.306	0.000	0.116–0.348	0.121–0.354

Number of samples is provided in brackets after the name of each population. lamp results are presented as the percentage of SNP alleles of wolf ancestry in autosomal chromosomes (at average) and in X chromosome (assessed only for individuals with sex known a priori and separately for males and females). We also report the number of admixed autosomal chromosomes, that is, having less than 90% of SNP alleles of wolf ancestry. The results of structure and admixture analyses are presented as the assignment probability of a given individual to the wolf cluster. “North American wolves” denote all North American wolf populations except Mexican wolves, which are presented separately. North American wolves were analysed in a separate lamp run rather than with Eurasian wolves.

To quantify uncertainty in ancestry estimates, we calculated 95% intervals for assignment probabilities from structure, as well as standard errors for the cluster membership estimates from admixture, which we used to calculate the 95% confidence intervals. For all individuals identified as F2/F3 back‐crosses, the upper limits of both intervals were below the range of values for nonadmixed individuals from the same geographic regions (Table S3). This supports our conclusion that these estimates reflect admixture rather than uncertainty of ancestry estimates.

In contrast, for most individuals with the admixture status classified as uncertain (see Table S3), the upper limits of both intervals were within the range of values for nonadmixed individuals. This does not preclude these individuals as being further generation back‐crosses, given the continuity of the assignment probability values from 0.75 to 1 in the wolf population (Figure S4). However, based on the existing genotyping data, we cannot reliably distinguish F4 or further generation back‐crosses from nonadmixed individuals.

The data set analysed also included coyotes, which at K = 3 were identified as a distinct genetic cluster alongside dogs and wolves (Figure S1). Coyotes had assignment probabilities to the dog cluster between 0 and 15% and to the wolf cluster between 0 and 7%. Coyotes hybridise with both dogs and North American grey wolves (vonHoldt et al., 2011), so this result could possibly reflect admixture. However, at K = 3, most wolves (all coming from Eurasia) were also shown to have some share of coyote ancestry, and therefore, it is more likely that these positive assignment probabilities reflect the common ancestry of wolves and coyotes. The inferred proportion of coyote ancestry in wolves declined with increasing K, but this was not the case for the inferred proportion of wolf and dog admixture in coyotes (Figure S1).

**Figure 4:**
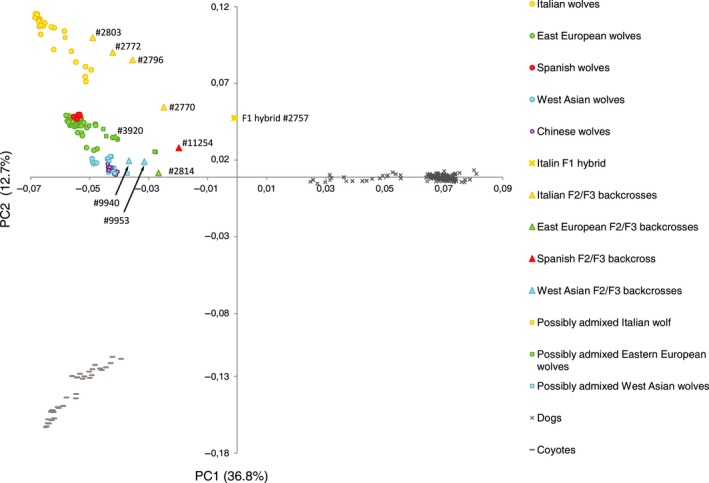
Principal component analysis (PCA) illustrating the extent of genetic diversification between Eurasian wolf populations and domestic dogs, and showing the position of the inferred wolf–dog F1 hybrid and recent back‐crosses relative to wolf and dog populations. Individuals labelled as “possibly admixed” are individuals with uncertain admixture status reported in Figure 3 and Table S2. The coyotes are included as out‐group. The PCA plot constructed without the coyotes is shown in Figure S2

In the PCA plot (Figure 4; Figure S2), canids identified as the F1 hybrid and F2/F3 back‐crosses based on structure and admixture analyses were distinct from their respective wolf populations and closer than other wolves to the dog cluster. The results of this analysis are described with more detail in Supporting Information, Part C.

### 3.2 Ancestry block analysis in Eurasian wolves

**Figure 5:**
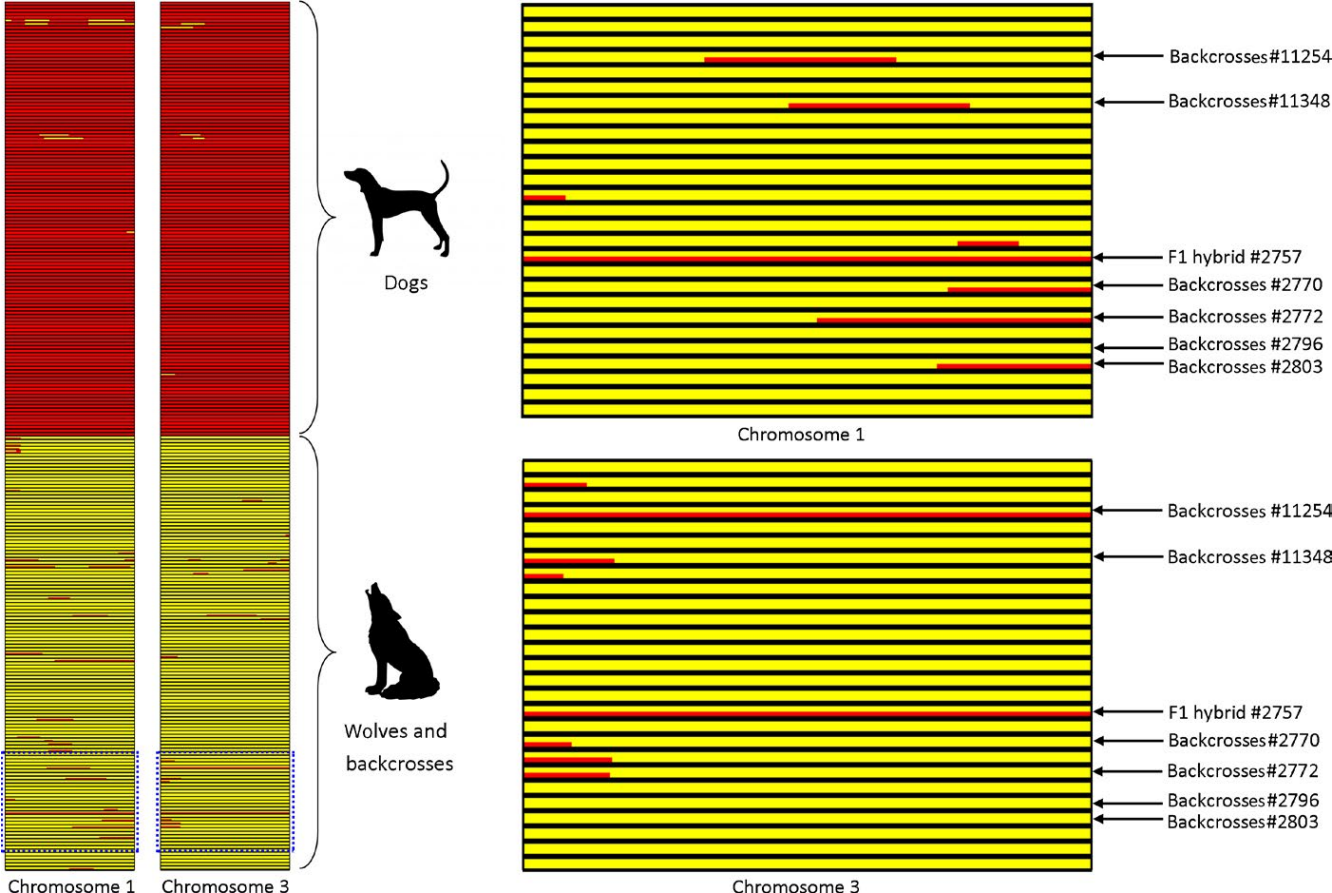
Results of ancestry block analysis in LAMP for two chromosomes, shown as an example. Dog ancestry is marked in red and wolf ancestry in yellow. Each row represents one individual, with dogs followed by wolves and admixed canids. A part of the graph is enlarged to show individual hybrids and back‐crosses. The ancestry plots for all 38 chromosomes are available in the Figure S3

The ancestry block analysis carried out using lamp identified two genetic clusters corresponding to wolves and dogs, and most individuals showed limited signs of admixed ancestry (Figure 5; Figure S3). Across all autosomal chromosomes, the mean percentage of SNP alleles of dog ancestry was less than 5% for each wolf except for the few individuals discussed below (see also Table S2). However, only 41 of 108 (38%) genotyped wolves had less than 10% of SNP alleles of dog ancestry on each chromosome, and only 25 of 108 (23%) wolves were completely free of small chromosomal blocks of assigned dog ancestry (i.e., had no SNP alleles of inferred dog ancestry). For European dog breeds, the mean percentage of SNP alleles of wolf ancestry was no higher than 0.3%. For ancient non‐European breeds, this percentage was between 0.2 and 11%.

LAMP results confirmed the admixture status of the F1 hybrid and all F2/F3 back‐crosses from European populations identified based on structure and admixture analyses (Figure 3; Table S2). The F1 hybrid (individual #2757) revealed 50% of SNP alleles of dog ancestry spanning all autosomal chromosomes, with one copy of each chromosome having dog ancestry and the other wolf ancestry (recombination was not inferred in the LAMP analysis). Individuals classified as F2/F3 back‐crosses based on structure and admixture results had 10–24% of SNP alleles of dog ancestry, consistent with the expected values (25% for F2 and 12.5% for F3 back‐crosses). Additionally, two other individuals from Eastern Europe had 6–7% of SNP alleles of dog ancestry, which was marginally outside the range for other wolves (0–5%). These individuals could be back‐crosses of further generations, but there is no strong support for this.

All individuals from Israel showed a relatively high percentage of SNP alleles of dog ancestry (5–14%), which is consistent with the structure and admixture analyses. Two individuals from Oman had only about 3% of SNP alleles of dog ancestry as inferred in LAMP, but had relatively high assignment probabilities to the dog cluster in structure and admixture analyses (Figure 3; Table S2).

We also counted the number of chromosomes for which the percentage of hybridisation‐derived SNP alleles (i.e., alleles of wolf ancestry in dogs, and alleles of dog ancestry in wolves and putative hybrids/back‐crosses) was higher than 10%. This number was low for European dog breeds (range: 0–1 chromosomes) and nonadmixed wolves (0–8 chromosomes). For individuals identified as F2/F3 back‐crosses, this number was considerably higher (12–23 chromosomes), while for individuals with uncertain admixture status, we observed intermediate values of 3–11 (Table S2).

The distribution of individuals with varying levels of admixed ancestry differed between wolves and dogs (Figure S4). In dogs, the majority of individuals had a very low percentage of SNP alleles of wolf ancestry, and only a small proportion of individuals, largely from ancient non‐European breeds, had a higher percentage of wolf‐derived alleles. In contrast, the Eurasian wolf populations represented a continuous range of admixture levels from individuals with no detectable dog ancestry to an individual with 24% of dog ancestry, which is consistent with a F2 back‐cross.

Due to the continuity of admixture levels, we were not able to distinguish between F2 and F3 back‐crosses or between further generations of back‐crosses and nonadmixed wolves (see also vonHoldt et al., 2013). However, the F1 hybrid could be identified without unambiguity, as this individual had one copy of each autosomal chromosome originating from wolves and one from dogs.

### 3.3 Ancestry block analysis for European wolves and European dog breeds

The results from a data set limited to European wolves and dog breeds of European origin only were in strong agreement with the results described above (Supporting Information Part B, Table S4). This demonstrates that the framework of the analysis (e.g., separate analysis for each wolf population versus joint analysis of different populations) does not affect the ability to detect hybrids and back‐crosses based on genomewide SNP data.

### 3.4 Ancestry blocks analysis in Eurasian wolves using PCadmix software

The inference of the dog admixture patterns in Eurasian wolves obtained from PCadmix was consistent with the inference from lamp. The average proportions of wolf ancestry in the assessed wolf data set were 0.958 and 0.955 based on lamp and PCAdmix analyses, respectively. There was a strong correlation between the dog ancestry proportions in individuals inferred using both methods (Spearman's rank correlation, rho = 0.875, *p *=* *2.62 × 10^−25^; see Figure S5A). There was also a strong correlation in the number of chromosomes identified as admixed using the two methods (Spearman's rank correlation, rho = 0.854, *p *=* *6.07 × 10^−23^; see Figure S5B). A chromosome was assumed to be admixed if it contained at least 10% of SNP alleles of dog ancestry assigned in lamp, or at least 10% of windows of dog ancestry assigned in PCadmix. The comparison of lamp and PCadmix further confirmed the continuous distribution of dog ancestry proportions in individuals from Eurasian wolf populations (Figure S5).

The individuals identified as F2/F3 hybrids based on the results from lamp, structure and admixture analyses had also similarly high dog ancestry proportions inferred in PCadmix, with the exception of individual #11254 from Spain (Figure 3; Table S2).

### 3.5 Geographic distribution of admixed individuals in Eurasia

Based on combined results from LAMP, structure and admixture analyses, we identified one F1 hybrid and nine F2/F3 back‐crosses among 108 wolves and 17 putatively admixed individuals from Eurasia (Figure 3). Most of these individuals (one F1 hybrid and seven F2/F3 back‐crosses) were found in European populations, which could be because all 17 putatively admixed individuals identified a priori came from Europe. F2/F3 back‐crosses were found in all European populations studied: two among 71 individuals from Eastern Europe, four among 20 individuals from Italy, and one among six individuals from the Iberian Peninsula. In the Arabian Peninsula, we identified two F2/F3 back‐crosses among 14 individuals. These numbers cannot be used to reliably assess the frequency of recently admixed individuals in the populations, because of small sample size and the presence of preselected putative hybrids in the sample. However, our results show that hybridisation is geographically widespread in Eurasian wolf populations.

### 3.6 Ancestry block analysis in North American wolves

Most North American wolves analysed here showed limited signs of dog admixture. With the exception of Mexican wolves and an individual from British Columbia (discussed below), each individual showed less than 0.6% of SNP alleles of dog ancestry (Table 1). Mexican wolves displayed 0.7 to 5.1% of SNP alleles of dog ancestry. An individual from Vancouver Island, British Columbia, had 21% of SNP alleles of dog ancestry and 16 chromosomes showing signs of admixture. This was the only individual among the North American wolves assessed that had an unambiguous signature of recent hybridisation. Among the wolves from North Canada there were two black individuals, and neither had detectable signs of dog ancestry.

European dog breeds showed low level of admixture with North American wolves (0.000–0.005), consistent with the corresponding result for Eurasian wolves. The level of North American wolf admixture detected in ancient non‐European dog breeds (0.000–0.043) was lower than the level of Eurasian wolf admixture (0.000–0.306).

### 3.7 Genetic differentiation between wolves and dogs at X chromosome

**Figure 6:**
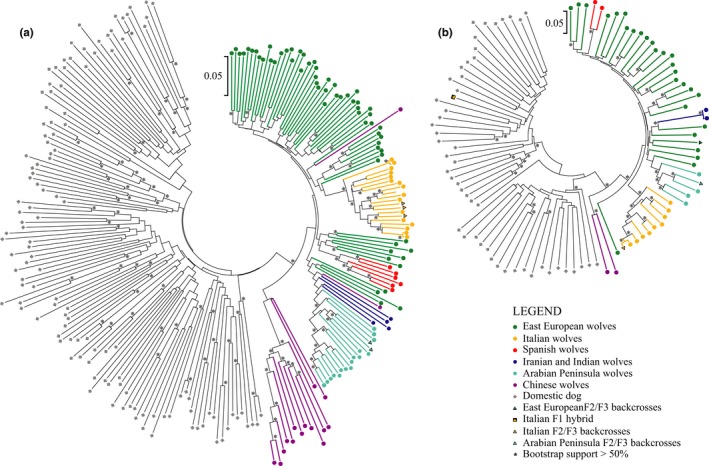
Evolutionary relationships of X chromosome haplotypes in (a) females and (b) males (right) inferred using the neighbour‐joining method. The distances were calculated using the p‐distance method. Bootstrap support is shown if higher than 50%

The neighbour‐joining tree of male X chromosome haplotypes clustered all wolves together with 67% bootstrap support and wolves from all regions, except China, with 97% support (Figure 6a). Chinese wolves were clustered together with 79% support. Italian wolves and Middle Eastern wolves (from Israel and Oman) formed two distinct subclades within the primary wolf clade with 99% and 87% bootstrap support, respectively. Haplotypes of Indian and Spanish wolves clustered with Eastern European wolves, but they were represented by only two individuals each. All F2/F3 back‐crosses identified based on autosomal data (Figure 3) grouped with their respective populations. However, the F1 hybrid (individual #2757) demonstrated X chromosome haplotype clustering with dog haplotypes.

The neighbour‐joining tree of female X chromosome haplotypes clustered all wolf haplotypes with 60% bootstrap support (Figure 6b). This tree is based on two haplotypes per female, which were reconstructed using fastPHASE. Wolves from China, the Middle East (Saudi Arabia and Israel), Italy and Spain formed four distinct clusters with 99%, 58%, 99% and 95% bootstrap support, respectively. Both haplotypes of one female from China did not group with other Chinese haplotypes, but instead one of them grouped with West Asian haplotypes, and the second with Eastern European haplotypes. All F2/F3 back‐crosses identified based on autosomal data (Figure 3) grouped with their respective populations. The X chromosome haplotype trees based on a data set limited to European wolves and dog breeds of European origin only were in strong agreement with the results described above, for both males and females (Supporting Information Part B, Figure S6).

lamp analysis of ancestry blocks on the X chromosome in females revealed no signs of dog ancestry in European and West Asian wolves, with the exception of two individuals from Europe with 12% and 4% of SNP alleles of dog ancestry, respectively (Table S5). In contrast, only one Chinese wolf lacked a signature of dog ancestry, while the other seven individuals analysed had 7–25% of SNP alleles of dog ancestry. Analysis of East Asian and Arctic dog breeds revealed 7–31% of SNP alleles of wolf ancestry, while this proportion was 2% in both Basenji and Dingo. European and West Asian breeds had the lowest proportion of 0–1.8% of SNP alleles originating from wolf admixture.

lamp analysis using X chromosome in males showed that the F1 hybrid #2757 had the entire X chromosome of dog ancestry. In two males from Eastern Europe, the estimated percentage of dog ancestry was 15% and 9%, respectively, which was considerably higher than that observed using autosome data (Table S5). In contrast, none of the males identified as F2/F3 back‐crosses based on autosomal chromosomes had detectable signature of dog ancestry in the X chromosome. No male dogs showed signature of wolf ancestry in the X chromosome (Table 1).

Analysis of population genetic structure at the X chromosome using admixture (carried out for females only) distinguished wolves and dogs as distinct groups at K = 2. Division into four groups (K = 4) was identified as the most likely genetic structure with dogs, Italian wolves, and Arabian wolves forming separate clusters, while wolves from other regions (Eastern Europe, Spain, Iran, India and China) were grouped together. Only one European wolf (from Italy) showed signs of dog admixture at the X chromosome. No admixture was detected in West Asia, but Chinese wolves had assignment probabilities to the dog cluster ranging from 6% to 19%. In European dog breeds, the assignment probabilities to the dog cluster were in the range 81–99%, whereas ancient breeds of Asian origin had probabilities of 51–79%.

Standard errors for the cluster membership estimates from admixture were higher for the X chromosome as compared with the autosomes. For all back‐cross females, the confidence intervals for the wolf cluster membership at the X chromosome included the value of 1.000 (Table S3). Therefore, these individuals could have both copies of their X chromosome originating from wolves, implying that they had male F1 hybrid ancestors originating from female wolf × male dog admixture.

### 3.8 Heterogeneity of dog ancestry proportions across the chromosomes in Eurasian wolves

The global CAnD test detected no significant heterogeneity in dog ancestry proportions across all autosomal chromosomes in the data set of all wolves studied (*p *=* *.072). For the data sets including both autosomal and X chromosome data for males and females, respectively, two autosomes had significantly lower proportions of dog ancestry as compared with the mean for all other autosomal chromosomes and the X chromosome (see Supporting Information, Part A, for details). We found no significant difference in dog ancestry proportions in the X chromosome as compared with the mean ancestry in autosomal chromosomes, in any of the data sets.

### 3.9 Heterozygosity, autozygosity and linkage disequilibrium in admixed individuals

Heterozygosity at autosomal chromosomes in admixed individuals from Italy (H_O_ = 0.23, H_E_ = 0.22) was considerably higher than in nonadmixed Italian wolves (H_O_ = 0.16, H_E_ = 0.16), and was within the range of Eastern European wolf populations (Table 2). Wolf populations from south‐western Europe and Saudi Arabia had lower heterozygosity levels compared to populations from Eastern Europe, Israel and China (Table 2).

**Table 2:** Heterozygosity and autozygosity in admixed individuals from Italy as compared with local populations of nonadmixed wolves from different parts of Eurasia

Local populations	H_O_	H_E_	No. of homozygous segments	Average length of homozygous segments (Kb)
Italian hybrids/back‐crosses	0.234	0.220	6.8	3,603
Italian wolves	0.161	0.155	1.6	2,449
Iberian wolves	0.173	0.169	1.5	1,902
East European wolves	0.214–0.235	0.219–0.263	2.2–6.5	1,771–5,142
Saudi Arabian wolves	0.179	0.156	7.4	6,445
Israeli wolves	0.215	0.222	6.1	6,695
Chinese wolves	0.221	0.235	2.8	4,154

H_O_, observed heterozygosity; H_E_, expected heterozygosity. Autozygosity is measured as average number of homozygous segments per individual, and their average length. East European wolves are represented by several local populations, and therefore, the range of values is provided.

Admixed individuals from Italy had a higher fraction of autozygous segments across all fragment sizes compared to Italian wolves and most other Eurasian wolf populations (Table 2). No autozygous segments were found in the F1 hybrid (individual #2757). Admixed individuals from Italy had lower LD levels (*r* ^2^ decayed below 0.5 at 387.5 Kb) than nonadmixed Italian wolves, where *r* ^2^ did not decay below 0.5 for the entire range of distances considered (up to 1 Mb). However, these admixed wolves still had higher LD levels than Iberian (257 Kb) and Eastern European wolves (2.5–10 Kb).

## 4 DISCUSSION

### 4.1 Detection of wolf–dog admixture based on genome‐wide SNP data

The application of genome‐wide SNP data has substantially improved resolution to detect admixture between canid species, compared to data generated using 10–30 microsatellite loci (vonHoldt et al., 2011). However, even with improved resolution, detecting hybridisation between grey wolves and their domesticated subspecies remains challenging, due to their recent shared common ancestry and the difficulty with a priori identification of nonadmixed individuals that are required as a reference in most methods of admixture analysis.

We carried out ancestry block analyses in Eurasian wolves in comparison with domestic dogs, applying a method implemented in the lamp software that did not require the use of nonadmixed reference populations. Under the assumption of a recent admixture in the past 10 generations (corresponding to about 30–40 years; Mech & Seal, 1987; Mech, Barber‐Meyer, & Erb, 2016) we were able to detect first‐generation wolf–dog hybrids, recent back‐crosses, and assess the overall level of admixture. The results we obtained when comparing Eurasian wolves and dog breeds of diverse origin were highly consistent with the results for a reduced data set consisting of European wolves and European dog breeds only. This finding suggests that the composition of wolf and dog data sets does not affect the ability to detect hybrids and recent back‐crosses based on genome‐wide SNP data. High consistency of the results from lamp with those from the PCadmix software, which required the use of reference populations, shows that admixed individuals can be detected independent of the choice of a particular analytical approach.

On average, the frequency of dog‐derived alleles in wolves was three times higher than the frequency of wolf‐derived alleles in the pure‐breed dogs we studied. If the alleles were incorrectly inferred as dog‐derived due to recent common ancestry of wolves and dogs, reference bias in the dominantly dog panel used to design the array (vonHoldt et al., 2010), or imperfect resolution of our method, a similar frequency of inferred wolf‐derived alleles should be expected in dogs (see also Supporting Information, Part D). The different proportions of alleles derived from hybridisation observed in the gene pools of dogs and wolves suggest more frequent introgression of dog alleles into the wolf gene pool than in the opposite direction. This is consistent with the expectations, given that we used pure‐bred dogs, which are unlikely to interbreed with wolves except as a result of a deliberate human action. The estimated levels of wolf alleles introgression to free‐ranging dog populations are likely to be higher.

### 4.2 Advantages and limitations of the data set used

The data set used in this study consisted of grey wolves sampled from across Eurasia, putative wolf–dog hybrids, and pure‐bred dogs. Through the use of pure‐bred dogs we ensured that we compare the wolf population (which admixture status was unknown prior to our analysis) with the nonadmixed dog population. Pure‐bred dogs can interbreed with wolves only via a deliberate human action, and we did not use breeds with a recent history of wolf admixture, such as Czechoslovakian wolf–dogs. In contrast, free‐ranging dog populations in Eurasia show signatures of introgression from grey wolves (Fan et al., 2016; Kopaliani et al., 2014; Pilot et al., 2015). The comparison with pure‐bred dogs allowed us to control the accuracy of our results, as we could expect limited levels of wolf admixture in pure‐bred dogs. This would not be possible if we compared two populations with unknown admixture levels (Eurasian wolves vs. free‐ranging dogs), and therefore pure‐bred dogs were more appropriate for our purpose.

On the other hand, free‐ranging dogs rather than pure‐bred dogs are the source of the introgression of dog alleles into wolf gene pool, and Eurasian free‐ranging dogs are a genetically distinct population instead of being an admixture of breeds (Pilot et al., 2015). Therefore, the comparison with free‐ranging dogs may result in higher levels of estimated dog introgression into wolf populations.

Another important feature of this data set was that it includes putative hybrids that were deliberately selected for genotyping from a larger data set. This allowed us to assess the accuracy of hybrid detection in previous studies based on a small number of microsatellite loci, and provided us with a sufficient number of admixed individuals to make conclusions regarding the mechanisms of admixture (e.g., the sex‐biased introgression). However, this data set cannot be used to assess the frequency of hybrids and recent back‐crosses in the wolf populations studied. For this purpose, a different sampling design will be required, without enrichment of the data set for putatively admixed individuals.

Our data set includes a relatively large sample of Italian wolves as compared with the sample sizes of other wolf populations. This could potentially affect the results of population structure analyses, showing that the Italian population is genetically distinct from other Eurasian populations (Figures 2 and 4). However, genetic distinctiveness of the Italian population was documented in a number of independent studies, and was shown to result from genetic drift during long‐term isolation (Lucchini, Galov, & Randi, 2004; Montana et al., 2017; Pilot et al., 2014). Our results are consistent with these earlier studies, and therefore we are confident that they are not an artefact of the uneven sample size.

### 4.3 Wolf–dog hybridisation in Eurasia

The ancestry block analysis unambiguously defined wolf and dog genetic clusters without any prior information about individuals’ origin, which confirms the results based on microsatellite loci analyses showing that Eurasian wolf populations are not hybrid swarms (Godinho et al., 2011; Hindrikson et al., 2012; Lorenzini et al., 2014; Randi & Lucchini, 2002). On the other hand, 62% of genotyped wolves carried small chromosomal blocks that were inferred to originate from dogs (see Supporting Information, Part E). This is consistent with the inference from genome re‐sequencing data, which suggests that most Eurasian grey wolves show some level of admixture with dogs (Fan et al., 2016). The presence of dog‐derived chromosomal blocks of varying size in the wolf gene pool in different regions studied suggests that introgressive hybridisation has occurred in distinct regions of Eurasia on a variety of timescales and is not solely a recent phenomenon.

This conclusion is also supported by the result of ancestry block analysis for two black‐coated individuals from Italy, showing no evidence of recent dog admixture. Both these individuals were heterozygous at the *CBD103* (beta‐defensin) gene and carried a dog‐derived allele linked to black colouration (Anderson et al., 2009; Caniglia et al., 2013), implying an ancient hybridisation event. This suggests that wolf–dog hybridisation in the Apennine Peninsula has occurred for many generations (in concordance with Randi et al., 2014), and that black wolves may be considered “pure” wolves with the exception of carrying the dog‐derived *CBD103* allele (although particular black individuals can be hybrids or recent back‐crosses). Our finding is consistent with the results of a recent study focused on wolf–dog hybridisation in Italy, which also detected a number of black‐coated wolves that showed no detectable signs of dog ancestry (Galaverni et al., 2017). The assumption that all black wolves derive from recent hybridisation, providing the rationale for eliminating them (Salvatori, 2015), is therefore incorrect. In fact, Italian canids that we genetically identified as recent back‐crosses were not black‐coated, hence, removing black wolves may not decrease the admixed ancestry of the population. This result shows that the elimination of individuals with atypical phenotypes is not always an appropriate management strategy for admixed populations.

Genetic introgression from a domesticated population into the wild ancestor is generally considered to be maladaptive as it compromises the genetic integrity of the wild species (Allendorf et al., 2001; Mallet, 2005), justifying management decisions to eliminate admixed individuals. However, in some cases, introgression of domestic gene variants may enhance adaptation. Anderson et al. (2009) showed that the mutation in *CBD103* gene linked to melanism exhibited a molecular signature of positive selection in North American grey wolves. Further studies showed that melanistic individuals which are heterozygous for the dog‐derived *CBD103* variant have a selective advantage over grey individuals in forested habitats (Coulson et al., 2011; Hedrick, Stahler, & Dekker, 2014; Stahler, MacNulty, Wayne, vonHoldt, & Smith, 2013). This example shows that hybridisation may provide wolf populations a way of acquiring new adaptations to a rapidly changing environment. Elimination of individuals possessing a single dog‐derived phenotypic trait may prevent such adaptations to be established in wolf populations. Therefore, management plans involving the lethal control of hybrids should consider both maladaptive and adaptive effects of admixture (see discussion in Wayne & Shaffer, 2016).

### 4.4 Continuity of dog ancestry proportions in Eurasian wolf populations and detection accuracy of back‐crosses

If hybridisation has occurred regularly throughout generations and has been followed by back‐crossing and gene introgression, we would expect that individuals with different proportions of dog ancestry, ranging between 0 and 0.25, would be present in the wolf population. This was indeed the case, as shown in Figure S4. Using similar logic, if hybridisation has been infrequent enough for the wolf and dog populations to retain their genetic distinctiveness, few individuals should be expected to have a share of dog ancestry ranging from 0.25 (corresponding to F2 back‐cross, i.e., offspring of a wolf and a F1 hybrid) and 0.5 (F1 hybrid), which can only be achieved from mating between recently admixed individuals. In our data set, we did not observe any individuals having a proportion of dog ancestry within this range.

Although F1 hybrids could be unambiguously identified based on ancestry block analysis, it was impossible to distinguish between F2 and F3 back‐crosses due to a lack of clear discontinuity between these two categories. There were also eight individuals in our data set that could have been F4 back‐crosses as they had >5% of estimated dog ancestry (the expected value for this generation of back‐crosses is 6.25%). However, some pure‐breed dogs displayed similar levels of admixture, which likely reflects more distant hybridisation events. Therefore, the precision of this analysis was insufficient to unambiguously detect back‐crossing at more distant levels.

The precision of back‐crosses detection was improved, however, compared with microsatellite loci analysis (e.g., Randi et al., 2014). Eight individuals from Eastern Europe and three from Italy which were previously identified as admixed based on microsatellite analysis, did not present as genetic outliers from their wolf populations based on the genome‐wide SNP data. This suggests that identification of admixed individuals based on a small number of microsatellite loci may be inaccurate beyond F1–F2 hybrids.

Genome‐wide SNP genotyping is still too expensive to be used routinely for management decisions, and typically requires high‐quality DNA extracts, precluding the use of noninvasive samples. We therefore suggest that both legal regulations and practical decisions regarding the management of admixed individuals clearly distinguish between F1 hybrids (which can be identified unambiguously based on a small number of genetic markers) and back‐crosses into wolf populations, which may be difficult to distinguish from nonadmixed wolves without an extensive genetic analysis.

### 4.5 Geographic patterns of admixture in Eurasian wolf populations

Individuals with recent admixed ancestry were detected in each of the European populations studied. In contrast, we detected no hybrids or recent back‐crosses in Iran, India and China, although the sample sizes from these countries were small. Contrasting patterns were found however in Chinese wolves in autosomes versus the X chromosome (see below). Wolves from the Arabian Peninsula showed signatures of dog admixture in eight of 14 individuals, with two individuals identified as F2/F3 back‐crosses. Given that no known admixed individuals from this region were included, this suggests that hybridisation has been particularly intense in this region. This finding is consistent with the inference of intense, bidirectional gene flow between Israeli wolves and dogs (Freedman et al., 2014), and the inference of gene flow from wolves to Saudi Arabian free‐ranging dogs (Pilot et al., 2015).

Taken together, these results show that wolf–dog hybridisation is geographically widespread in Eurasia, but its frequency may vary considerably between regions. Earlier genetic studies on European wolves based on microsatellite loci, estimated the frequencies of admixed individuals at 5.6% in the Iberian Peninsula (Pacheco et al., 2017), 5% in Italy (Verardi et al., 2006) and 9.8% in Bulgaria (Moura et al., 2014). These varying estimates may suggest differences in hybridisation rate between regions, but could also result from differences in methodological approaches between the studies. A comparative assessment of hybridisation levels would require the use of the same genetic markers and analytical methods for different geographic regions, an even sample coverage and an unbiased sampling process, without preferential sampling of putative hybrids. The knowledge of large‐scale geographic patterns of hybridisation may help understand whether different methods of wolf management (regulated hunting, unregulated hunting, full protection) affect the frequency of hybridisation.

### 4.6 Admixture patterns inferred from the X chromosome data

The X chromosome haplotypes of all back‐crosses identified in this study grouped within the wolf cluster. This pattern suggests sex‐biased introgression of dog alleles into wolf populations, with male dogs having a higher contribution than females. In Chinese wolves, all but one female had positive assignment probabilities (up to 25%) to the dog cluster at the X chromosome, but no admixture was detected in these individuals based on autosomal chromosomes. This result suggests an introgression of dog X chromosome haplotypes following an ancient hybridisation event, and possibly selection acting upon genes on the X chromosome. Representatives of East Asian dog breeds (Chow Chow and Akita) also had positive assignment probabilities to the wolf cluster, suggesting that the hybridisation resulted in a bidirectional introgression of X chromosome haplotypes between dogs and wolves.

The X and Y chromosome patterns imply that the only F1 hybrid identified in our sample set, Italian male #2757, was the offspring of a female dog and a male wolf. This individual was identified a priori as admixed based on its atypical phenotype (as reported in the ISPRA database “putative hybrid or dog‐like”; the details of the phenotype or photos are not available) and its structure‐based assignment probability to the Italian wolf cluster was 0.44 based on 39 autosomal microsatellite loci (Randi et al., 2014). We found that the X chromosome haplotype of this male clustered with dogs, and Randi et al. (2014) found that he carried Y chromosome haplotype YH17 (inferred from four microsatellite loci data), which is commonly found in Italian wolves. This result indicates that this individual was the offspring of a male wolf. However, most previously described cases of natural wolf–dog hybridisation involved female wolves that mated with male dogs (Andersone et al., 2002; Godinho et al., 2011; Iacolina et al., 2010; Vilà et al., 2003). A review of wolf–dog hybridisation patterns worldwide concluded that mating between male wolves and female dogs is less frequent and/or it is rarely followed by back‐crossing of the resulting hybrids into the wolf population (Leonard et al., 2014). Nevertheless, examples of hybrids having male wolf × female dog ancestry are known from earlier studies (Hindrikson et al., 2012), and the hybrid we have identified here unequivocally represents such case.

We also found signs of female‐mediated introgression of dog alleles in two males from Eastern Europe, which displayed relatively high estimated percentage of dog ancestry (15% and 9%, respectively) in the X chromosome, but lower percentage in the autosomes. Yet, all but one individuals identified as F2/F3 back‐crosses based on the analysis of autosomes had 100% of X chromosome SNP alleles matched to those defining wolf ancestry. This result suggests that mating of female wolves with male dogs may be more favourable for introgression of dog alleles into wolf populations. However, the test of heterogeneity in dog ancestry proportions between the X chromosome and autosomal chromosomes for the entire data set of Eurasian wolves was nonsignificant, implying either the lack of sex bias in the introgression, or insufficient power. Further studies are needed to clarify this, given important implications for the management of admixing populations. If the introgression of dog alleles is male‐biased, it could be limited by sterilisation of free‐ranging male dogs, but this would not be sufficient if the introgression is not sex‐biased.

### 4.7 Wolf–dog hybridisation in North America

In contrast to Eurasian wolves, most North American grey wolves showed no signal of admixture with dogs. In Mexican wolves, we found SNP alleles matching dog ancestry, but their frequency (1–5%) was too small to make conclusions regarding the admixture status of this population. This finding does not imply conservation concerns regarding the genetic integrity of the Mexican wolf population, but indicates the need for more extensive research into possible past hybridisation of Mexican wolves with other canids.

Among the North American wolves studied, only one individual, from Vancouver Island, British Columbia, was identified as a recent back‐cross (probably F3). The Vancouver Island population has been shown previously to have experienced hybridisation with dogs, which likely occurred at early stages of recolonisation of the island in the 1970–1980s (Munoz‐Fuentes, Darimont, Paquet, & Leonard, 2010). This result suggests that strong demographic fluctuations and range contractions/expansions promote cross‐breeding with dogs. Eurasian wolf populations have experienced strong bottlenecks and range fluctuations, some of which are well documented either based on direct demographic inference (Boitani, 2003), or genetic analyses (e.g., Fan et al., 2016; Montana et al., 2017; Pilot et al., 2014). Moreover, Eurasian wolf populations have been sympatric with dogs for a longer period, given that the dog domestication occurred in Eurasia (Freedman & Wayne, 2017). Both factors may contribute to the higher frequency of alleles originating from dogs in gene pools of in Eurasian versus North American wolves.

The sympatric occurrence of coyotes in large parts of the North American wolf range may be of importance as well, as wolves may show preference towards mating with coyotes rather than dogs (vonHoldt, Kays, Pollinger, & Wayne, 2016; vonHoldt et al., 2011). Although North American wolves show signatures of ancient hybridisation with dogs (Anderson et al., 2009), studies documenting recently admixed individuals are rare (Munoz‐Fuentes et al., 2010). In consistent with our results, whole‐genome sequence data showed signatures of recent admixture with dogs in Eurasian wolves, but not in North American wolves (Fan et al., 2016). Understanding the reason underlying this difference in hybridisation patterns may help develop effective strategies to manage admixing wolf populations.

Our analysis also found low levels of admixture from North American wolves in domestic dogs breeds of both European and non‐European origin, with the highest level estimated at 4%. By comparison, the admixture analysis between Eurasian wolves and domestic dogs indicated considerable input (up to 11%) of wolf‐derived variants into ancient breeds, particularly breeds in East Asian and Arctic origin. This implies that the past hybridisation event(s) resulting in wolf admixture in ancient breeds occurred in Eurasia rather than North America (see Supporting Information, Part D, for further discussion of wolf admixture in dogs).

The large differences in the frequency of dog‐derived alleles in the Eurasian versus North American wolf populations provide evidence that dog admixture inferred in lamp does not represent background noise produced by the method. With the exception of Mexican wolves and one individual from Vancouver Island discussed above, the maximum share of dog ancestry detected in North American wolves was 0.006. This value may be considered as the maximum rate of erroneous assignment of dog origin to small chromosomal segments using lamp. In the Eurasian populations studied, 54% of individuals had an estimated proportion of dog ancestry exceeding this value. If small chromosomal segments attributable to dogs in Eurasian wolves were false positives produced by lamp, they should have been detected in North American wolves with a similar frequency as in Eurasian wolves, which was not the case.

### 4.8 Heterozygosity, autozygosity and linkage disequilibrium in admixed individuals

Italian wolves have low heterozygosity and high LD as a result of long‐term isolation and a bottleneck (Montana et al., 2017; Pilot et al., 2014). Italian canids identified as wolf–dog hybrids or back‐crosses had considerably higher heterozygosity and lower LD than pure Italian wolves. Although hybridisation is generally expected to increase LD, in this case it had an opposite effect, due to Italian wolves displaying particularly long stretches of LD (Pilot et al., 2014).

A study of the Scandinavian wolf population showed that the most heterozygous individuals establish themselves as breeders (Bensch et al., 2006). If this is a general rule, back‐crossed individuals may have a selective advantage over pure wolves in populations with low heterozygosity levels, such as the Italian population. Interestingly, back‐crossed Italian individuals carried a higher fraction of autozygous segments across all fragment sizes than pure wolves, suggesting that hybridisation was followed by mating with related individuals in subsequent generations. This conclusion is supported by the work of Caniglia et al. (2013) who chronicled a recent hybridisation event followed by breeding between close relatives in a single Italian pack of wolves.

### 4.9 Conclusions and management implications

We detected the presence of small blocks of dog ancestry in the genomes of 62% wolves sampled from all Eurasian populations analysed, suggesting that hybridisation has occurred in different parts of Eurasia, throughout multiple generations, and is not solely a recent phenomenon. Nevertheless, the wolf populations have maintained a distinct genetic profile from dogs, suggesting that hybridisation and back‐crossing have occurred at a low frequency.

We found that two melanistic wolves who carried a dog‐derived allele at a beta‐defensin locus, displayed no signs of recent admixed ancestry. In contrast, some individuals identified a priori as “pure” wolves were shown to be F2 or F3 back‐crosses. This result implies that phenotype alone cannot be reliably used to distinguish between back‐crosses and nonadmixed individuals. Our data also suggest that Eurasian wolf populations represent a continuum of genotypes from “pure” wolves to F2 back‐crosses. This makes the definition of genetically “pure” wolves ambiguous, and raises questions about appropriate management of back‐crossed individuals, as they may be too difficult to identify and too numerous to be removed from wolf populations (see Wayne & Shaffer, 2016).

Back‐crossed individuals are typically integrated into wolf packs, and disruption of pack structure due to culling may enhance hybridisation (Moura et al., 2014). Therefore, even if admixed individuals could be unambiguously identified, their removal may be ineffective and could eventually generate more hybrids. The efficient management of admixed populations should be focused, instead, on reducing the factors which cause hybridisation, such as small population size, the presence of free‐ranging dogs and unregulated hunting (Moura et al., 2014). Also, increasing the proportion of natural wolf habitats and their natural prey may enhance retention of wolf genomic elements by natural selection (Wayne & Shaffer, 2016). We also recommend that any documents regulating legal status of admixed canids should distinguish between F1 hybrids and back‐crosses into wild populations. Although this study was specifically focused on grey wolves and domestic dogs, our conclusions are applicable to any case where hybridisation with a domesticated species may affect the genetic integrity of a closely related wild species.

## DATA ARCHIVING

This project was based on the analysis of data published in vonHoldt et al. (2010), which can be accessed at http://genome-mirror.bscb.cornell.edu/cgi-bin/hgGateway (see “SNPs” track under the Variations and Repeats heading).
