# Supplementary material for: Widespread, long‐term admixture between grey wolves and domestic dogs across Eurasia and its implications for the conservation status of hybrids
Source: Evol Appl. 2018 Mar 8;11(5):662–80. doi: 10.1111/eva.12595 (PMC5978975; doi:10.1111/eva.12595)
Supplement: Supplementary file 1 [file EVA-11-662-s001.pdf]

SUPPORTING INFORMATION

Widespread, long-term admixture between grey wolves and domestic dogs across Eurasia and its implications for the conservation status of hybrids

Małgorzata Pilot, Claudia Greco, Bridgett M. vonHoldt, Ettore Randi,

Włodzimierz Jędrzejewski, Vadim E. Sidorovich, Maciej K. Konopiński,

Elaine A. Ostrander, Robert K. Wayne

Part A

Heterogeneity of dog ancestry proportions across the chromosomes in Eurasian wolves

We applied the Chromosomal Ancestry Differences (CAnD) test (McHugh et al. 2016) to assess whether there are significant differences in dog ancestry contributions among the chromosomes in Eurasian wolves. Important features of this method are that (1) it accounts for the lack of independence of ancestries in different chromosomes within individuals and (2) does not require specification of the admixture history of the population tested (McHugh et al. 2016). We used the CAnD test to assess whether there are significant differences in dog ancestry proportions between each of the autosomes and the X chromosome, using a Bonferroni correction for multiple testing. We compared the ancestry proportions inferred in LAMP for Eurasian wolves, including admixed individuals, using the following datasets: (1) LAMP output for autosomal chromosomes only, for all individuals, (2) LAMP output for autosomal chromosomes and the X chromosome for females, (3) LAMP output for autosomal chromosomes and the X chromosome for males, (4) LAMP output for autosomal chromosomes and the combined X chromosome outputs for males and females.

For the dataset of dog ancestries at autosomal chromosomes for all individuals studied, the global CAnD test detected no significant heterogeneity in dog ancestry

proportions across all chromosomes (P=0.072). The tests assessing differences in dog ancestry proportions in each chromosome as compared with the mean ancestry of all remaining autosomal chromosomes all gave non-significant results after the Bonferroni correction (P values ranging from 0.1 to 1).

For the dataset of dog ancestries at autosomal chromosomes and the X chromosome for females, the global CAnD test detected significant heterogeneity in dog ancestry proportions across chromosomes (P=0.016). We found no significant difference in dog ancestry proportions in the X chromosome as compared with the mean ancestry in autosomal chromosomes (P=1 after the Bonferroni correction). However, we found that the chromosome 31 had a significantly lower proportion of dog ancestry as compared with the mean for all other chromosomes (P=0.031 after the Bonferroni correction).

For the dataset of dog ancestries at autosomal chromosomes and the X chromosome for males, the global CAnD test detected no significant heterogeneity in dog ancestry proportions across all chromosomes (P=0.742). We found no significant difference in dog ancestry proportions in the X chromosome as compared with the mean ancestry in autosomal chromosomes (P=1 after the Bonferroni correction). However, we found that the chromosome 2 had a significantly lower proportion of dog ancestry as compared with the mean for all other chromosomes (P=0.003 after the Bonferroni correction).

For the dataset of dog ancestries at autosomal chromosomes and the X chromosome for females and males combined, the global CAnD test detected no significant heterogeneity in dog ancestry proportions across all chromosomes (P=0.121). The tests assessing differences in dog ancestry proportions in each chromosome (including the X chromosome) as compared with the mean ancestry of all remaining autosomal chromosomes all gave non-significant results after the Bonferroni correction (P values ranging from 0.2 to 1).

Part B

Analysis of wolf-dog hybridisation based on a dataset of European wolves and dog breeds of European origin

To assess whether a choice of a particular set of wolf and dog populations affects the results of hybridisation analysis, we repeated the analyses using ADMIXTURE, STRUCTURE and LAMP for a smaller dataset consisting of European wolves and dog breeds of European origin. This dataset consisted of 147 individuals: 54 Eastern European wolves, 19 Italian wolves, 6 Iberian wolves, 17 putative wolf-dog hybrids (9 from Eastern Europe and 8 from Italy), and 51 dogs of different breeds (1 individual per modern dog breed).

ADMIXTURE and STRUCTURE analyses were carried out using the same input settings as in the analysis of the original dataset described in the main text. In the LAMP analysis, we assumed mixture proportion 0.65:0.35. The mixture proportion is the proportion of alleles deriving from two ancestral populations (grey wolves and domestic dogs in this case). We estimated this proportion as the frequency of wolves and putative admixed individuals *versus* dogs in the dataset. This was based on a conservative assumption that the set of putative admixed individuals can include back-crosses to the wolf population besides F1 hybrids, and therefore these individuals will have higher proportion of wolf alleles than dog alleles in their genomes. We also made test runs with mixture proportions 0.75:0.25 and 0.5:0.5. The 0.75:0.25 proportion produced very similar results as compared with 0.65:0.35 proportion, while 0.5:0.5 proportion resulted in all wolves and dogs being admixed. We used the recombination rate of 1e-8, and all the other parameters were the same as in the analysis of the original dataset described in the main text.

The analysis of X chromosome data was carried out using the same methods as in the analysis of the original dataset described in the main text.

Results

Analysis of autosomal chromosome data

Both ADMIXTURE and STRUCTURE identified dogs versus wild canids as most distinct subdivision (at K=2). At K=3, Italian wolves were identified as the third distinct group. The assignment of the putative hybrids was consistent between the three programs and the two datasets. Italian canid 2757 was assigned as a F1 hybrid, with 47-50% assignment to the dog cluster. Four other Italian individuals were assigned to the dog cluster with probabilities 10-15% (Supporting Table S4), suggesting that they were back-crosses. The remaining three Italian individuals were assigned to the Italian wolf population with over 99% probability. One of the putative Eastern European hybrids, individual 2814 from Kirov, Russia was assigned to the dog cluster with 15-16% probability depending on the program used. Other putative hybrids from Eastern Europe had assignment probabilities to the dog cluster lower than 4%, which was within the range for the pure wolves.

Two individuals that were assumed *a priori* to be pure wolves, individual 11254 from Spain and 11348 from Ukraine had assignment probabilities to the wolf population 0.838-0.846 and 0.869-0.876, which was outside the range for other wolves (0.925-1.000), but within the range for back-crosses (0.838-0.907), so they were inferred to be back-crosses as well. Two other individuals, 10103 from Belarus and 11315 from Ukraine had assignment probabilities to the wolf population 0.914-0.921 and 0.908-0.916, respectively (Supporting Table S4). They could be further generation of back-crosses, but this inference is uncertain.

The ancestry block analysis carried out in LAMP was performed without assuming *a priori* which individuals represent non-admixed, ancestral populations. Non-admixed individuals were identified in the analysis in an analogous way as distinct populations are identified in STRUCTURE. Across all but one autosomal chromosomes, the mean percentage of SNPs assigned to chromosomal blocks of wolf ancestry was less than 2% for each dog, and

the mean percentage of SNPs assigned to chromosomal blocks of dog ancestry was less than 6% for each wolf except for few admixed individuals (see Supporting Table S4). For chromosome 35, the percentage of SNPs assigned to chromosomal blocks of wolf ancestry was 20% or more for each dog, and thus this chromosome was excluded from further summary statistics.

Italian canid 2757 was assigned as a F1 hybrid, as it had at average 50% of SNPs assigned to chromosomal blocks of dog ancestry, and this value was consistent among different chromosomes (range of dog ancestry: 46-59%). In 29 out of 37 chromosome pairs in this individual, one of the chromosomes was entirely assigned dog ancestry and another one wolf ancestry. Four putative hybrids from Italy, one from Kirov, Russia, and two putative pure wolves from Spain and Ukraine had 12-23% of SNPs assigned to chromosomal blocks of dog ancestry. These individuals were assigned as back-crosses, which was consistent with the results from ADMIXTURE and STRUCTURE (Supporting Table S4). Additionally, three other individuals from Eastern Europe (from Belarus, Ukraine and Bulgaria) had 7-9% of SNPs assigned to chromosomal blocks of dog ancestry, which was marginally outside the range for other wolves (0-6%). For the first two of these individuals, this result was consistent with the results from ADMIXTURE and STRUCTURE (Supporting Table S4). These individuals could be back-crosses of further generations, but their exact admixture status was uncertain.

We also counted the number of chromosomes for which the percentage of mis-assigned SNPs (i.e. SNPs assigned to blocks of wolf ancestry in dogs, and blocks of dog ancestry in wolves and putative hybrids/back-crosses) was lower than 90%, excluding chromosome 35 from this count. This number was low for dogs (range: 0-2) and non-admixed wolves (range: 0-8). For individuals identified as back-crosses, this number was substantially higher than in non-admixed wolves (range: 14-23), and the three individuals from Eastern Europe with the

uncertain status had intermediate values (range: 11-12).

All these results were highly consistent with the corresponding results obtained for a larger dataset that also included Asian wolves and dog breeds of non-European origin (see Supporting Table S2). All hybrids and back-crosses identified in the main analysis (Supporting Table S2) were also identified here (Supporting Table S4). This allows us to conclude that the choice of a particular set of populations representing two “pure” subspecies in the admixture analysis (using the methods applied here) does not affect our ability to detect hybrids and recent back-crosses. However, in this study we encountered difficulties with distinguishing further-generations (F4/F5) back-crosses from “pure” wolves. For such analysis, the presence of direct representatives of particular populations that are subject to admixture may be required, given that some wolf populations (like Arabian Peninsula wolves) may have high “background” levels of admixture reflecting ancient/historical hybridisation.

Analysis of X chromosome data

The neighbour-joining tree of male X chromosome haplotypes distinguished wolf and dog haplotypes with 98% bootstrap support (Supporting Figure S6A). Italian wolf haplotypes formed a distinct subclade within the wolf clade with 92% support. Putative hybrids from Italy grouped with the Italian wolves, except for individual 2757, which had X chromosome haplotype clustering in the dog clade. Putative hybrids from Eastern Europe were all grouped with the Eastern European wolves, except for individual 2814 from Kirov, Russia, that had a basal position in the wolf clade.

The neighbour-joining tree of female X chromosome haplotypes distinguished wolf haplotypes from dog haplotypes with 99% bootstrap support (Supporting Figure S6B). Both Italian and Spanish wolf haplotypes formed distinct subclades within the wolf clade with

99% bootstrap support. Putative hybrids from Italy and Eastern Europe grouped with the Italian and Eastern European wolves, respectively.

LAMP analysis of ancestry blocks in X chromosome in females showed that European wolves have no more than 7% of SNP alleles of dog ancestry, and European dog breeds have no more than 11% of chromosomal blocks of wolf ancestry. Only two individuals (both from Italy) assigned as F2/F3 back-crosses were females. One of them had 4% of SNP alleles of dog ancestry, which was well within the range for the pure wolves. The second individual, however, had 15% of SNP alleles of dog ancestry (Supporting Table S4).

The results of the X chromosome ancestry block analysis in males had a worse resolution: the percentage of mis-assigned SNP alleles was 0-20% in European wolves, and 8-41% in dogs (Supporting Table S4). One of the male back-crosses had 100% of X-chromosome SNP alleles of wolf ancestry, implying that it had no female dog ancestor. In two other back-crosses, the percentage of SNP alleles assigned to chromosomal blocks of dog ancestry was 14 and 15%. The F1 hybrid #2757 had 88% of SNP alleles of dog ancestry, well within the range for the dogs.

Analysis of population genetic structure in ADMIXTURE for X chromosome data for the dataset consisting of European wolves, putative hybrids, and dogs (females only) indicated K=3 as the most likely genetic structure, with Italian wolves, Eastern European wolves and dogs forming separate groups. The assignment probability of wolves to the dog cluster was 0.00001 for each individual, except individual #10121 from Russia with the assignment probability 0.036. The assignment probabilities of dogs to the dog cluster were in the range 0.695-0.999.

For comparison, the same set of individuals was also analysed for genetic structure at autosomal chromosomes. The assignment probabilities of wolves to the dog cluster were in the range 0.000-0.041, except individual #10121 with the assignment probability 0.066. Two

female backcrosses, #2796 and #2803, had the assignment probabilities to the dog cluster 0.191 and 0.107, respectively, while the corresponding values for the X chromosome were 0.050 and 0.000. The assignment probabilities of dogs to the dog cluster were in the range 0.909-0.999.

Part C

Results of the Principal Component Analysis

PCA analysis of wolves, dogs, wolf-dog hybrids and coyotes discriminated between dogs and wild canids on the first axis (PC1), and separated the wolves from the coyotes at the second axis (PC2) (Figure 3). The clusters of dogs, wolves and coyotes were clearly distinct, and Italian wolves formed a distinct sub-cluster, while other wolf populations displayed relatively low differentiation.

In the PCA plot constructed without coyotes, the first axis (PC1) discriminated between dogs and wild canids, while the second (PC2) separated the Italian wolves from other Eurasian wolves (Supporting Figure S2). In both PCA plots (with and without coyotes), the same individuals were identified as outliers from population clusters.

European canids identified as the F1 hybrid and F2/F3 backcrosses based on STRUCTURE and ADMIXTURE analyses were distinct from their respective wolf populations and closer than other wolves to the dog cluster (Figure 3). In addition, three individuals from Eastern Europe (two not identified as admixed in STRUCTURE and ADMIXTURE and one with uncertain admixture status) did not cluster with the Eastern European wolf population. Canids from the Arabian Peninsula identified as F2/F3 backcrosses based on STRUCTURE and ADMIXTURE analyses were distinct from their population in the plot including the coyotes (Figure 3), but in the plot without the coyotes they were not clearly distinct (Supporting Figure S2).

Part D

Signatures of wolf admixture in domestic dogs

Past studies on wolf-dog hybridization focused largely on the signatures of hybridisation in wolf populations, but Kopaliani et al. (2014) showed that hybridisation also has a considerable effect on local dog populations, with over 10% of shepherd dogs sampled in rural areas of Georgia in the Caucasus having a detectable wolf ancestry. Consistent with this finding, Ardalan et al. (2011) suggested that one of the mtDNA haplogroups present in native dogs from Iran, Afghanistan and Turkey originates from introgression from wolves. Analysis of whole-genome sequences of modern wolves and dogs also indicated past bi-directional gene flow between them (Freedman et al., 2014).

Although this study was focused on the signatures of dog admixture in grey wolf populations, it also provided novel information regarding wolf admixture in domestic dogs. Pure-breed dogs analysed in this study are unlikely to have interbred with wolves throughout recent generations (we did not analyse breeds where such interbreeding was deliberate and documented, such as Czechoslovakian wolfdog). The ancestry block analysis indicated low level (up to 0.3%) of Eurasian wolf admixture in dog breeds of European origin (i.e. breeds developed in Europe or breeds developed elsewhere from dogs of European origin), but considerable level of admixture (up to 11%) in ancient breeds of non-European origin, and in particular in East Asian and Arctic breeds. Arctic dog breeds trace a part of their ancestry to ancient Siberian wolves (Skoglund et al., 2015), and similar ancient or historical hybridisation events could have also occurred in East Asia, but it is unlikely that hybridisation would have occurred in the last 10 generations (as assumed in the LAMP analysis) without being documented. This suggests that the LAMP analysis cannot be used to accurately estimate the time since admixture, if it is more distant than 3-4 generations. The

analysis which includes the data from the ancestral populations (i.e. non-admixed wolves and dogs) may improve the precision of such estimates.

The wolf admixture levels in pure-breed dogs on the X chromosome were considerably higher as compared to autosomal chromosomes, suggesting greater contribution to the dog genome from female wolves than male wolves. This result is consistent with a study by Sundqvist et al. (2006), showing a sex bias in the origin of modern dog breeds, with fewer males than females contributing genetically. Greater contribution of females implies greater sharing of maternal than paternal lineages between dogs and wolves. This is consistent with mtDNA lineage sharing between the two subspecies, and with a very clear distinction between them based on autosomal microsatellites (Parker et al. 2004), genome-wide SNPs (vonHoldt et al. 2010, Pilot et al. 2015), and nuclear genome sequences (Fan et al. 2016).

The inferred admixture levels at X chromosome were higher in "ancient" non-European breeds as compared with European breeds, mirroring the pattern observed at autosomal chromosomes. The ADMIXTURE analysis typically indicated East European or East Asian wolves as sources of wolf admixture in dogs, but two breeds originating in the Middle East, Basenji and Saluki, showed signatures of admixture with wolves from the Arabian Peninsula. This suggests that cross-breeding with wolves occurred multiple times and in different locations.

The inferred past admixture between the ancient breeds and Eurasian wolves could possibly explain the apparent distinctiveness of these breeds from modern European breeds (e.g. Parker et al. 2004; vonHoldt et al., 2010; Pilot et al., 2015). However, the ancient breeds maintain their basal position in the dog phylogeny when gene flow is explicitly accounted for in the phylogenetic reconstruction (Pilot et al., 2015), implying that the wolf admixture alone cannot explain genetic differentiation between ancient and modern breeds.

References

Ardalan, A., Kluetsch, C. F., Zhang, A. B., Erdoğan, M., Uhlén, M., Houshmand, M., ..., Savolainen, P. (2011). Comprehensive study of mtDNA among Southwest Asian dogs contradicts independent domestication of wolf, but implies dog-wolf hybridization. Ecology and Evolution, 1, 373–385.

Fan, Z., Silva, P., Gronau, I., Wang, S., Armero, A. S., Schweizer, R. M., …Wayne, R. K. (2016). Worldwide patterns of genomic variation and admixture in gray wolves. Genome Research, 26, 163-173.

Freedman, A. H., Gronau, I., Schweizer, R. M., Ortega-Del Vecchyo, D., Han, E., Silva, P. M.,…Novembre J. (2014). Genome sequencing highlights the dynamic early history of dogs. PLoS Genetics, 10, e1004016.

Kopaliani, N., Shakarashvili, M., Gurielidze, Z., Qurkhuli, T., Tarkhnishvili, D. (2014). Gene Flow between Wolf and Shepherd Dog Populations in Georgia (Caucasus). Journal of Heredity, 105, 345-353.

Parker, H.G., Kim, L.V., Sutter, N. B., Carlson, S., Lorentzen, T. D., … Ostrander E. A. (2004). Genetic structure of the purebred domestic dog. Science, 304, 1160–1164.

Pilot, M., Malewski, T., Moura, A. E., Grzybowski, T., Oleński, K., Ruść, A., … Bogdanowicz, W. (2015). On the origin of mongrels: evolutionary history of free-breeding dogs in Eurasia. Proceedings of the Royal Society B: Biological Sciences 282, 20152189.

Skoglund P., Ersmark E., Palkopoulou E., Dalen L. (2015). Ancient wolf genome reveals an early divergence of domestic dog ancestors and admixture into high-latitude breeds. Current Biology, 25, 1–5.

Sundqvist A.-K., Bjornerfeldt S., Leonard J. A., Hailer F., Hedhammar A, Ellegren H., Vila C. (2006). Unequal Contribution of Sexes in the Origin of Dog Breeds. Genetics, 172, 1121–1128.

vonHoldt, B., Pollinger, J. P., Lohmueller, K. E., Han, E., Parker, H. G., Quignon, P., … Wayne, R. K. (2010). Genome-wide SNP and haplotype analyses reveal a rich history underlying dog domestication. Nature, 464, 898-903.

Part E

Comment on the evidence for past admixture in Eurasian wolves

The ancestry block analysis in LAMP indicated that only 38% of genotyped Eurasian wolves were free of small chromosomal blocks that were inferred to originate from dogs. This may be incorrectly interpreted as evidence for very high rate of ongoing hybridisation in Eurasia. Therefore, it is important to stress that the presence of a small proportion of small chromosomal blocks of dog ancestry in an individual genome indicates that such individual had a dog ancestor in its genealogy many generations ago, rather than implying a recent hybridisation event. In a similar example, genomes of humans of Eurasian origin carry 1.5-2.1% of alleles originating from the Neanderthals (Prüfer et al. 2014), which obviously does not imply ongoing frequent interbreeding, but is a signature of past hybridisation that was occurring in the Late Pleistocene. In our study, we identified only ten individuals resulting from recent hybridisation events: one F1 hybrid and nine F2/F3 backcrosses. Therefore, our results do not imply that wolf-dog hybridisation in Eurasia occurs currently at high frequency, but that is has been occurring in different parts of Eurasia on multiple timescales and is not solely a recent phenomenon.

It should be also considered whether the inferred signatures of dog ancestry in wolves and wolf ancestry in dogs can result from the recent shared ancestry between wolves and dogs rather than from hybridisation. We found that the average frequency of dog-derived alleles in wolves was three times higher than the frequency of wolf-derived alleles in pure-breed dogs we studied. If the alleles were incorrectly inferred as dog-derived due to recent common ancestry of wolves and dogs, a similar frequency of inferred wolf-derived alleles should be expected in dogs. This disproportion can be explained by differences in the frequency of back-crossing of hybrids into wolf versus dog populations, given that we studied pure-breed dogs, whose breeding patterns are strictly controlled by humans.

We also found large differences in the frequency of dog-derived alleles in Eurasian vs North American wolf population. This inference is consistent with large number of previous studies reporting recent hybridisation in different Eurasian populations (see references in the main text), but scarcity of such reports from North America (Munoz-Fuentes et al., 2010). This could be potentially explained by the common ancestry domestic dogs share only with Eurasian wolf populations, given that the dog originated in Eurasia (Freedman & Wayne, 2017). This explanation is however unlikely, given that the divergence time of Eurasian and North American wolves was estimated to occur only 285-1,565 years (95% CI) before the estimated divergence of dogs from Eurasian wolves (Fan et al., 2016).

In addition, we also inferred considerably higher frequency of wolf-derived alleles in ancient non-European breeds as compared with breeds of European origin. This can be explained by ancient hybridisation event(s) between local dog and wolf populations. If the inferred wolf-derived alleles were an artefact of the recent shared ancestry between wolves and dogs, similar frequency of such alleles should be expected in all dog breeds independent of their geographic origin. We also observed a large variation in the frequency of dog-derived alleles between individual Eurasian wolves, ranging from 0 to 0.5, and the frequency distribution (Figure 6B) is consistent with hybridisation followed by introgression. Finally, our interpretation of the results is consistent with the inference based on whole-genome data (Freedman et al., 2014; Fan et al., 2016).

References

Fan, Z., Silva, P., Gronau, I., Wang, S., Armero, A. S., Schweizer, R. M., …Wayne, R. K. (2016). Worldwide patterns of genomic variation and admixture in gray wolves. Genome Research, 26, 163-173.

Freedman, A. H., Gronau, I., Schweizer, R. M., Ortega-Del Vecchyo, D., Han, E., Silva, P. M.,…Novembre J. (2014). Genome sequencing highlights the dynamic early history of dogs. PLoS Genetics, 10, e1004016.

Freedman, A. H., Wayne, R. K. (2017). Deciphering the origin of dogs: from fossils to genomes. Annual Review of Animal Biosciences, 5, 281-307.

Munoz-Fuentes, V., Darimont, C.T., Paquet, P.C., Leonard, J.A. (2010). The genetic legacy of extirpation and re-colonization in Vancouver Island wolves. Conservation Genetics, 11, 547 – 56.

Prüfer, K., Racimo, F., Patterson, N., Jay, F., Sankararaman, S., Sawyer, S., ..., Paabo, S. (2014). The complete genome sequence of a Neandertal from the Altai Mountains. Nature, 505, 43–49.

Part F

STRUCTURE and ADMIXTURE analyses: optimum number of clusters and "convex" behaviour

STRUCTURE and ADMIXTURE results were inconsistent regarding the optimum number of genetic clusters (K value). In STRUCTURE analysis, the highest delta K value was for K=2 and the second highest for K=3, whereas the highest estimated likelihood was for K=7. In ADMIXTURE the lowest cross-validation error was obtained for K=6. The genetic clusters identified at K=6 were consistent between STRUCTURE and ADMIXTURE, with the exception of the clustering of dogs. STRUCTURE grouped modern European breeds in one cluster and East Asian and Arctic breeds in the second cluster, whereas ADMIXTURE grouped the boxer and related breeds in one cluster and all other breeds in the second cluster. At K=7, STRUCTURE identified the same dog clusters as ADMIXTURE at K=6, and Spanish wolves as an additional cluster. In contrast, ADMIXTURE at K=7 did not identify additional clusters of wolves, but instead identified three clusters of dogs: one cluster grouping East Asian and Arctic breeds, the second cluster grouping the boxer and related breeds, and the third cluster grouping other European breeds. Although there was no consistency between the methods with regard to an optimum K value, the differences were in clustering patterns in dogs, and did not affect our inference.

ADMIXTURE was previously shown to display a "convex" behaviour, i.e. a tendency to produce ancestry estimates that are biased away from 50% towards either 0% or 100% (Engelhardt & Stephens, 2010). In our study, we identified only one first generation hybrid. For this individual, LAMP assigned exactly 50% of alleles at each chromosome to dogs and 50% to wolves, resulting in the overall admixture proportion of 0.5. In comparison, the wolf admixture proportion for this individual inferred from ADMIXTURE was 0.549, and from STRUCTURE 0.545. We have not found any other individuals with the dog ancestry within the range of 0.25 – 0.75, implying that admixture between hybrid or recent backcrosses is rare, and therefore this individual had to be a F1 hybrid. This suggests that there was indeed a bias away from 50% in ADMIXTURE and STRUCTURE results. However, for other admixed individuals, we did not find a systematic bias towards 100% in ADMIXTURE and/or STRUCTURE as compared with LAMP (Figure 3, Supporting Table S2).

Reference

Engelhardt, B.E., Stephens, M. (2010). Analysis of population structure: A unifying framework and novel methods based on sparse factor analysis. PLoS Genetetics, 6(9), e1001117.

Supporting Tables

Supporting Table S1. The allele frequency divergence among populations estimated in STRUCTURE at K=8. We report these estimates for 8 inferred genetic clusters, because at these K value most geographically distinct wolf populations were inferred.

	Italian wolves	Eastern European and Spanish wolves	Arabian wolves	Central Asian wolves	East Asian wolves	European dog breeds	non-European dog breeds	coyotes
Italian wolves	-							
Eastern European and Spanish wolves	0.079	-						
Arabian wolves	0.145	0.095	-					
Central Asian wolves	0.093	0.043	0.094	-				
East Asian wolves	0.108	0.059	0.116	0.065	-			
European dog breeds	0.148	0.101	0.151	0.099	0.110	-		
non-European dog breeds	0.129	0.080	0.133	0.081	0.088	0.079	-	
coyotes	0.136	0.085	0.139	0.089	0.102	0.145	0.122	-

Table note: High divergence values observed between some wolf populations (e.g. between Italian wolves and other wolf populations) are due to strong genetic drift resulting from isolation and bottlenecks (see Pilot et al. 2014).

Reference

Pilot, M., Greco, C., vonHoldt, B.M., Jędrzejewska, B., Randi, E., Jędrzejewski, W., … Wayne, R. K. (2014). Genome-wide signatures of population bottlenecks and diversifying selection in European wolves. Heredity, 112, 428–442.

Supporting Table S2. Hybrids and back-crosses identified in Eurasian wolf populations based on the ancestry blocks analysis in LAMP and PCADMIX, and the analysis of population genetic structure in STRUCTURE and ADMIXTURE (assuming K=2), using autosomal chromosome data.

			LAMP		PCADMIX				
**ID**	**Region**	**Prior assess-ment of admix-ture**	**Assign-ment proba-bility**	**No. admixed auto-somes**	**Assign-ment proba-bility**	**No. admixed auto-somes**	**STRUC-TURE**	**ADMIX-TURE**	**Inferred ancestry**
2757	Italy	admixed	0.500	38	0.478	38	0.545	0.549	F1 hybrid
2770	Italy	admixed	0.843	15	0.876	18	0.892	0.896	back-cross
2772	Italy	admixed	0.832	21	0.819	22	0.867	0.869	back-cross
2796	Italy	admixed	0.790	19	0.785	21	0.834	0.837	back-cross
2803	Italy	admixed	0.840	17	0.841	17	0.893	0.895	back-cross
2814	Russia	admixed	0.894	12	0.927	9	0.837	0.843	back-cross
11348	Ukraine	pure	0.904	13	0.880	14	0.859	0.865	back-cross
11254	Spain	pure	0.760	23	0.992	1	0.825	0.832	back-cross
10103	Belarus	pure	0.929	11	0.920	11	0.902	0.907	uncertain
11315	Ukraine	pure	0.936	10	0.927	11	0.901	0.907	uncertain
2788	Latvia	admixed	0.943	8	0.938	7	0.928	0.934	uncertain
2725	Italy	pure	0.946	8	0.944	8	0.977	0.977	uncertain
11342	Russia	pure	0.987	3	0.980	3	0.974	0.981	uncertain*
9953	Israel	pure	0.861	16	0.832	18	0.790	0.797	back-cross
9940	Israel	pure	0.914	12	0.884	12	0.823	0.831	back-cross
9938	Israel	pure	0.934	9	0.916	10	0.861	0.869	uncertain
9959	Israel	pure	0.939	8	0.918	12	0.852	0.859	uncertain
9945	Israel	pure	0.939	10	0.920	13	0.872	0.879	uncertain
9937	Israel	pure	0.947	8	0.920	12	0.866	0.873	uncertain
3076	Oman	pure	0.969	3	0.938	6	0.843	0.851	uncertain
3077	Oman	pure	0.968	4	0.949	5	0.833	0.841	uncertain

Table note: LAMP results are presented as the percentage of SNP alleles of wolf ancestry in autosomal chromosomes (at average) and in X chromosome (assessed only for individuals with sex known *a priori*, and separately for males and females). We also report the number of admixed autosomal chromosomes, i.e. chromosomes having more than 10% of SNP alleles of dog ancestry (estimated in

LAMP) or more than 10% of 20-SNP windows of dog ancestry (estimated in PCADMIX). The results of STRUCTURE and ADMIXTURE analyses are presented as the assignment probability of a given individual to the wolf cluster. The ancestry of admixed individuals listed in the last column is inferred based on these results.

Supporting Table S3. Standard error estimates for the population assignment values (q) to the wolf cluster from STRUCTURE and ADMIXTURE analysis (assuming K=2) for hybrids and back-crosses identified in Eurasian wolf populations.

		STRUCTURE (AUTOSOMES)		ADMIXTURE (AUTOSOMES)		ADMIXTURE (X CHROMOSOME, FEMALES)		
**ID**	**Region**	**Q-VALUE**	**95% PI**	**Q-VALUE**	**95% CI**	**Q-VALUE**	**95% CI**	**Inferred ancestry**
2757	Italy	0.545	0.534-0.555	0.549	0.538-0.561			F1 hybrid
2770	Italy	0.892	0.883-0.902	0.896	0.869-0.923			back-cross
2772	Italy	0.867	0.859-0.874	0.869	0.837-0.902			back-cross
2796	Italy	0.834	0.826-0.842	0.837	0.806-0.868	0.938	0.832-1.000	back-cross
2803	Italy	0.893	0.885-0.900	0.895	0.866-0.925	0.999	0.990-1.000	back-cross
2814	Russia	0.837	0.827-0.847	0.843	0.824-0.863			back-cross
11348	Ukraine	0.859	0.851-0.867	0.865	0.840-0.890			back-cross
11254	Spain	0.825	0.814-0.836	0.832	0.817-0.847			back-cross
10103	Belarus	0.902	0.894-0.909	0.907	0.884-0.931			uncertain
11315	Ukraine	0.901	0.893-0.908	0.907	0.885-0.930			uncertain
2788	Latvia	0.928	0.921-0.935	0.934	0.913-0.956			uncertain
2725	Italy	0.977	0.972-0.981	0.977	0.961-0.993			uncertain
11342	Russia	0.974	0.968-0.980	0.981	0.968-0.993			uncertain*
9953	Israel	0.790	0.781-0.799	0.797	0.769-0.825	0.965	0.866-1.000	back-cross
9940	Israel	0.823	0.815-0.832	0.831	0.807-0.854			back-cross
9938	Israel	0.861	0.853-0.869	0.869	0.849-0.888			uncertain
9959	Israel	0.852	0.844-0.860	0.859	0.837-0.881			uncertain
9945	Israel	0.872	0.864-0.880	0.879	0.860-0.898	0.977	0.913-1.000	uncertain
9937	Israel	0.866	0.858-0.874	0.873	0.855-0.892	0.999	0.941-1.000	uncertain
3076	Oman	0.843	0.835-0.852	0.851	0.835-0.867			uncertain
3077	Oman	0.833	0.824-0.841	0.841	0.825-0.857			uncertain

Supporting Table S4. Hybrids and back-crosses identified in European wolf populations based on the chromosome blocks analysis in LAMP, and the analysis of population genetic structure in STRUCTURE and ADMIXTURE (assuming K=2). These analyses were carried out for a dataset consisting of European wolves and dog breeds of European origin. LAMP results are presented as the percentage of SNPs assigned to chromosomal blocks of wolf origin in autosomal chromosomes (at average) and in X chromosome (performed only for individuals with sex known a priori, and separately for males and females), and the number of admixed autosomal chromosomes, i.e. having less than 90% of SNPs assigned to chromosomal blocks of wolf origin. The results of the structure-based analyses are presented as the probability of assignment of a given individual to the wolf cluster. The origin of an individual is inferred based on these results.

		LAMP results						
**ID**	**Region**	**autosomes**	**No. admixed auto-somes**	**chr X females**	**chr X males**	**STRUCTURE**	**ADMIXTURE**	**Inferred origin**
2757	Italy	0.500	37		0.118	0.545	0.549	F1 hybrid
2770	Italy	0.832	19			0.900	0.904	back-cross
2772	Italy	0.816	22		0.858	0.877	0.880	back-cross
2796	Italy	0.776	21	0.852		0.842	0.846	back-cross
2803	Italy	0.829	17	0.964		0.904	0.907	back-cross
2814	Russia	0.830	23		0.854	0.838	0.847	back-cross
11254	Spain	0.794	34			0.838	0.846	back-cross
11348	Ukraine	0.885	14		1.000	0.869	0.876	back-cross
10103	Belarus	0.914	12		1.000	0.914	0.921	uncertain
11315	Ukraine	0.932	11		0.866	0.908	0.916	uncertain
range for dogs		0.004-0.019	0-1	0.027-0.105	0.085-0.411	0.001-0.080	0.000-0.089	
range for wolves		0.940-0.999	0-8	0.933-0.998	0.805-1.000	0.925-1.000	0.935-1.000	

Supporting Table S5. Comparison of the wolf ancestry proportions in hybrids and back-crosses at the autosomal chromosomes and the X chromosome, based on the inference from LAMP and ADMIXTURE (assuming K=2).

			LAMP results			ADMIXTURE results		
ID	Region	Prior assessment of admixture	auto-somes	chr X females	chr X males	auto-somes	chr X females	Inferred ancestry
2757	Italy	admixed	0.500		0.000	0.549		F1 hybrid
2770	Italy	admixed	0.843			0.896		back-cross
2772	Italy	admixed	0.832		1.000	0.869		back-cross
2796	Italy	admixed	0.790	0.877		0.837	0.938	back-cross
2803	Italy	admixed	0.840	1.000		0.895	0.999	back-cross
2814	Russia	admixed	0.894			0.843		back-cross
11254	Spain	pure	0.760			0.832		back-cross
11348	Ukraine	pure	0.904		1.000	0.865		back-cross
10103	Belarus	pure	0.929		1.000	0.907		uncertain
11315	Ukraine	pure	0.936		0.849	0.907		uncertain
11342	Russia	pure	0.987		0.915	0.981		uncertain
2788	Latvia	admixed	0.943		1.000	0.934		uncertain
2725	Italy	pure	0.946			0.977		uncertain
9953	Israel	pure	0.861	1.000		0.797	0.965	back-cross
9940	Israel	pure	0.914		1.000	0.831		back-cross
9938	Israel	pure	0.934		1.000	0.869		uncertain
9959	Israel	pure	0.939		1.000	0.859		uncertain
9945	Israel	pure	0.939	1.000		0.879	0.977	uncertain
9937	Israel	pure	0.947	1.000		0.873	0.999	uncertain
3076	Oman	pure	0.969		1.000	0.851		uncertain
3077	Oman	pure	0.968		1.000	0.841		uncertain

Supporting Figures

Supporting Figure S1. Genetic differentiation between regional populations of wolves and domestic dogs inferred using the programs (A) STRUCTURE and (B) ADMIXTURE, assuming the number of inferred clusters (K) from 2 to 6. Coyote – *Canis latrans*; Dog – *Canis lupus familiaris*: **EU**ropean breeds, **NON EU**ropean breeds; Wolf – *Canis lupus*: from left to right **SP**ain, **IT**aly, **E**astern **E**urope (Bulgaria, Croatia, Greece, Turkey – European part, Slovakia, Poland, Lithuania, Belarus, Ukraine, Russia), **AS**ia (Israel, Arabia, Oman, Iran, India, China).

LEGEND

▼ F1 hybrid

▼ Backcross: from left to right: 1 individual from Spain, 4 from Italy, 1 from Ukraine, 1 from Russia and 2 from Israel.

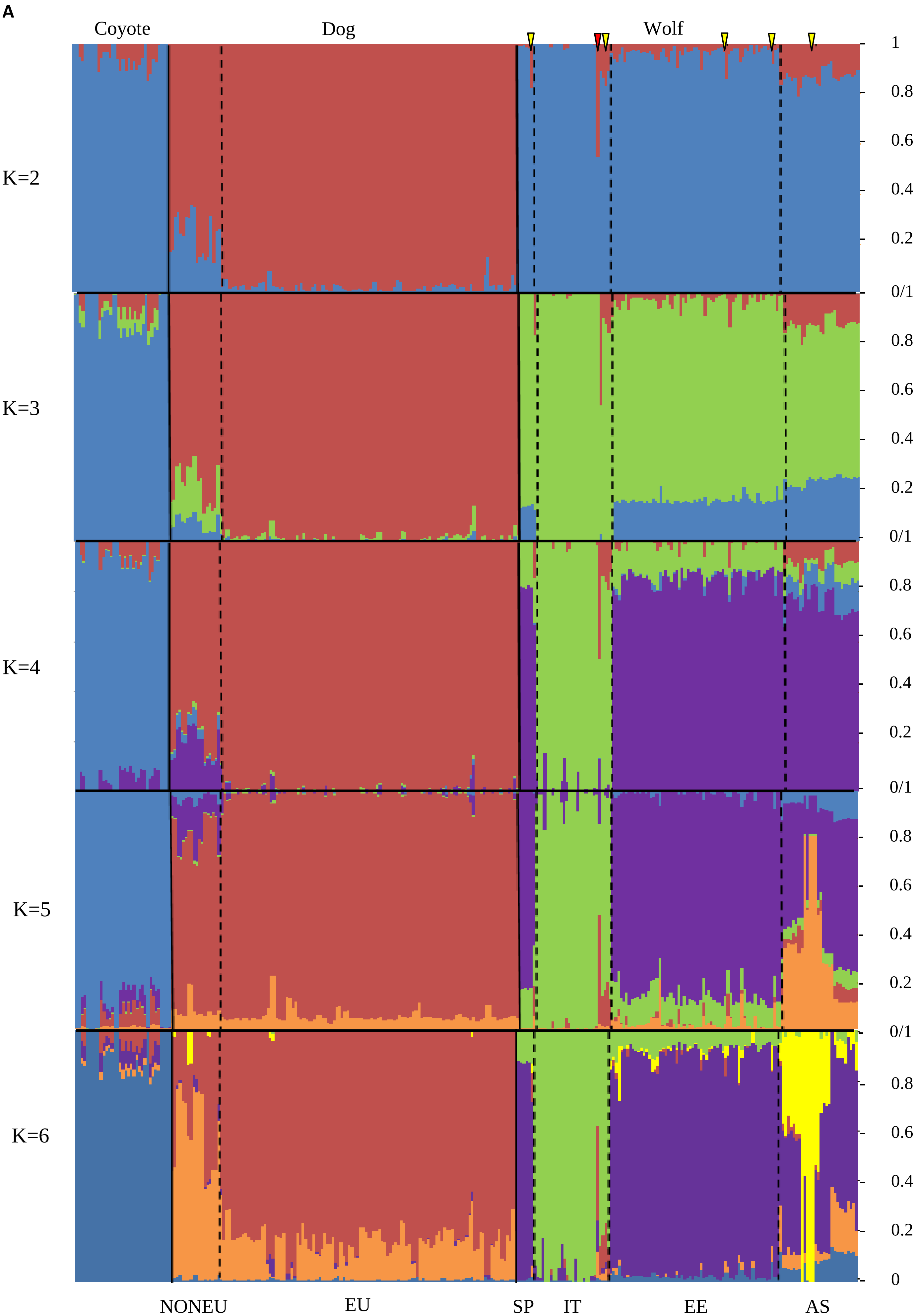
A
Coyote
Dog
Wolf
K=2
K=3
K=4
K=5
K=6
1
0.8
0.6
0.4
0.2
0/1
0
NONEU
EU
SP
IT
EE
AS

B

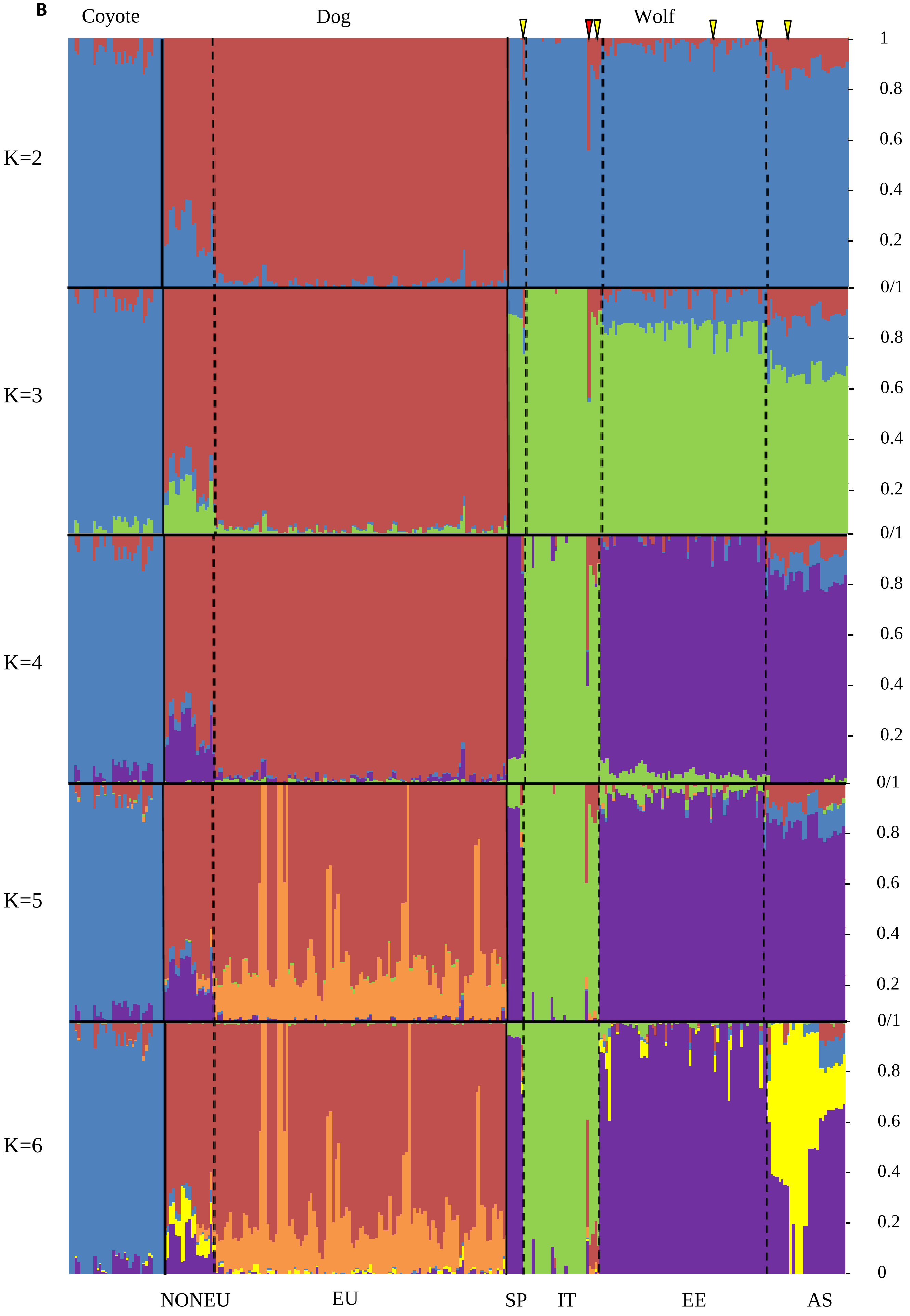
Coyote
Dog
Wolf
K=2
K=3
K=4
K=5
K=6
1
0.8
0.6
0.4
0.2
0/1
0
NONEU
EU
SP
IT
EE
AS

Supporting Figure S2. Principal component analysis (PCA) illustrating the extent of genetic diversification between Eurasian wolf populations and domestic dogs, and showing the position and ID numbers of the inferred wolf-dog hybrids and recent back-crosses relative to wolf and dog populations. Individuals labelled as "possibly admixed" are individuals with uncertain admixture status reported in Table 1.

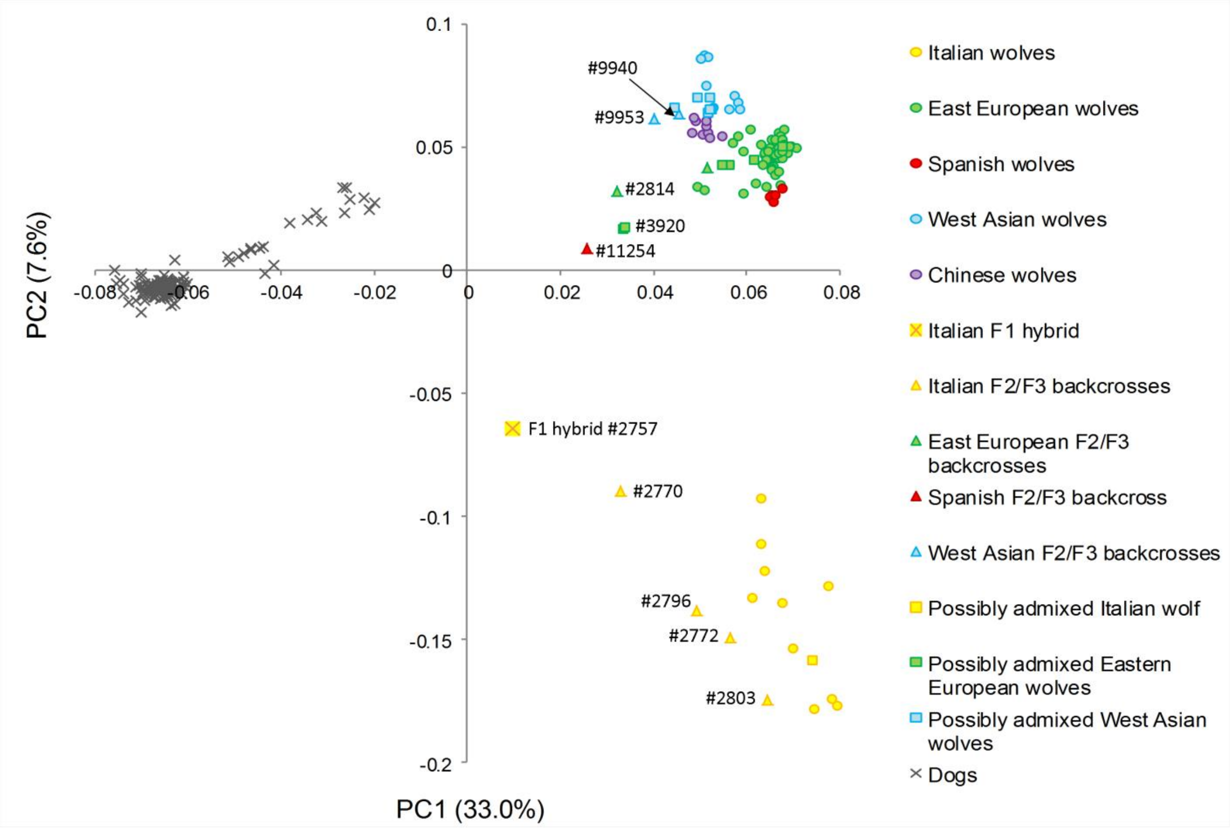

Supporting Figure S3. Results of ancestry block analysis from LAMP for 38 autosomal chromosomes. Dog ancestry is marked in red and wolf ancestry in yellow. Each row represents one individual, with dogs followed by wolves and admixed canids.

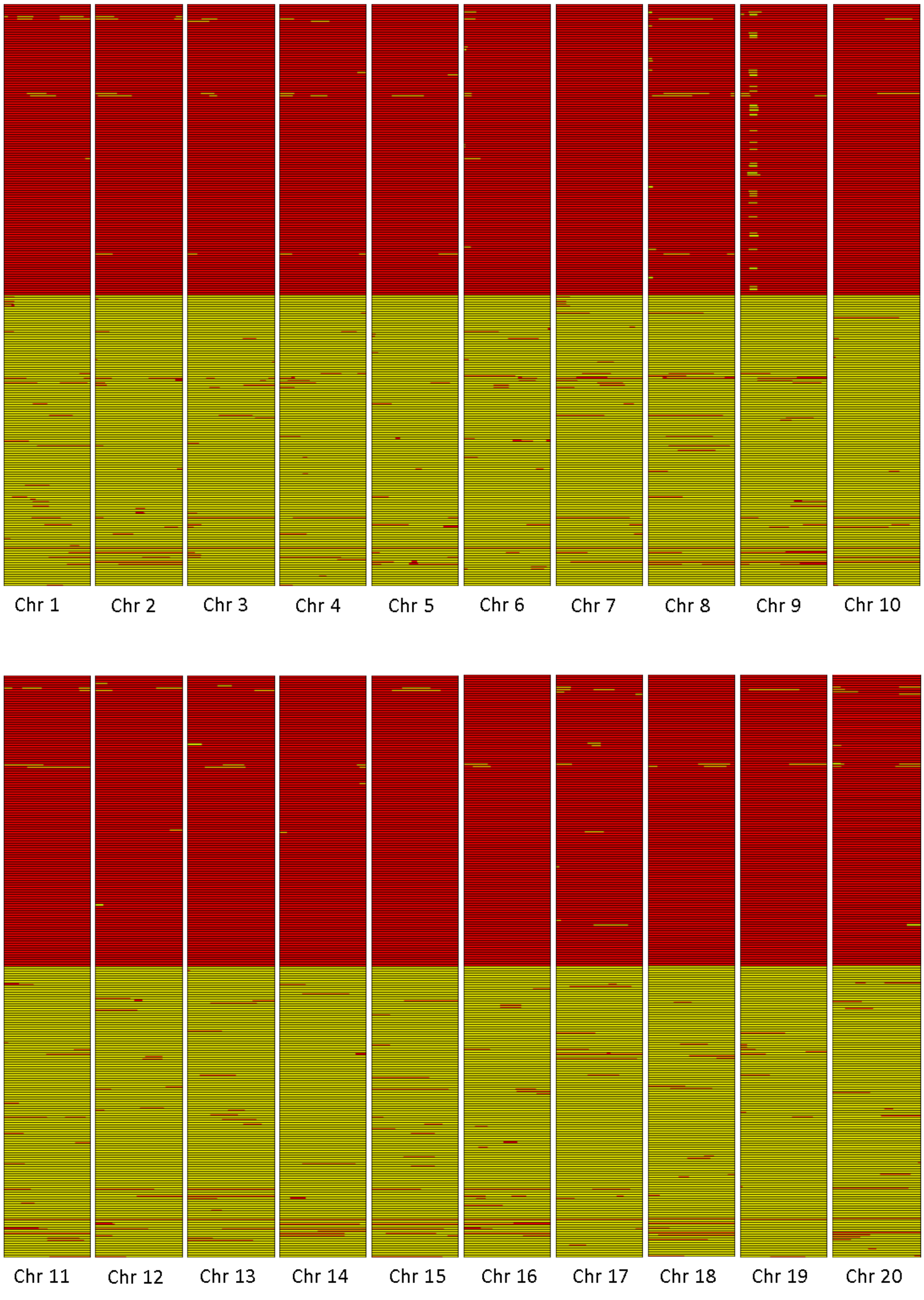

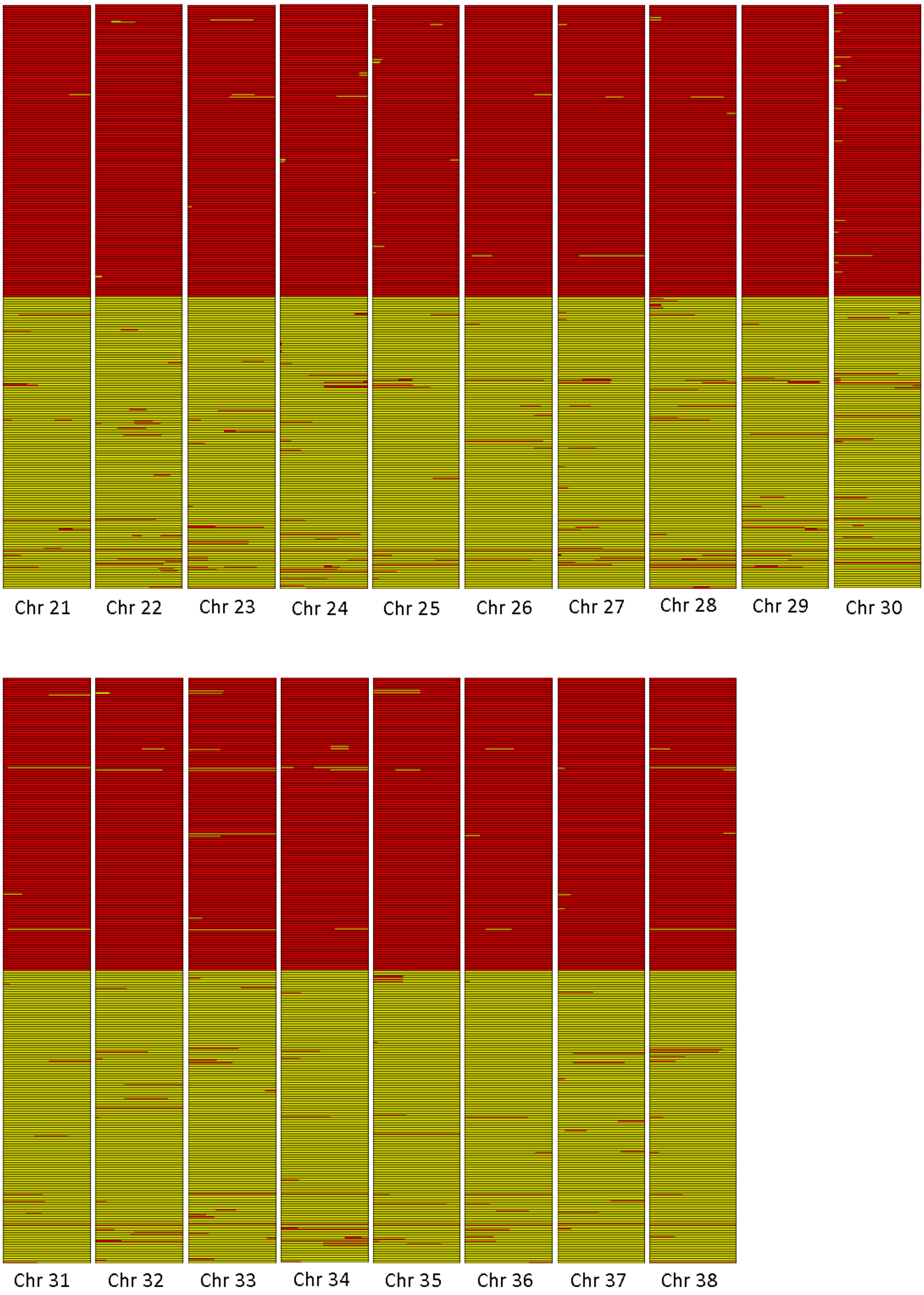
Chr 21
Chr 22
Chr 23
Chr 24
Chr 25
Chr 26
Chr 27
Chr 28
Chr 29
Chr 30
Chr 31
Chr 32
Chr 33
Chr 34
Chr 35
Chr 36
Chr 37
Chr 38

Supporting Figure S4. Distribution of individuals with different levels of admixed ancestry in (A) dogs, (B) wolves. Both plots are based on 124 individuals.

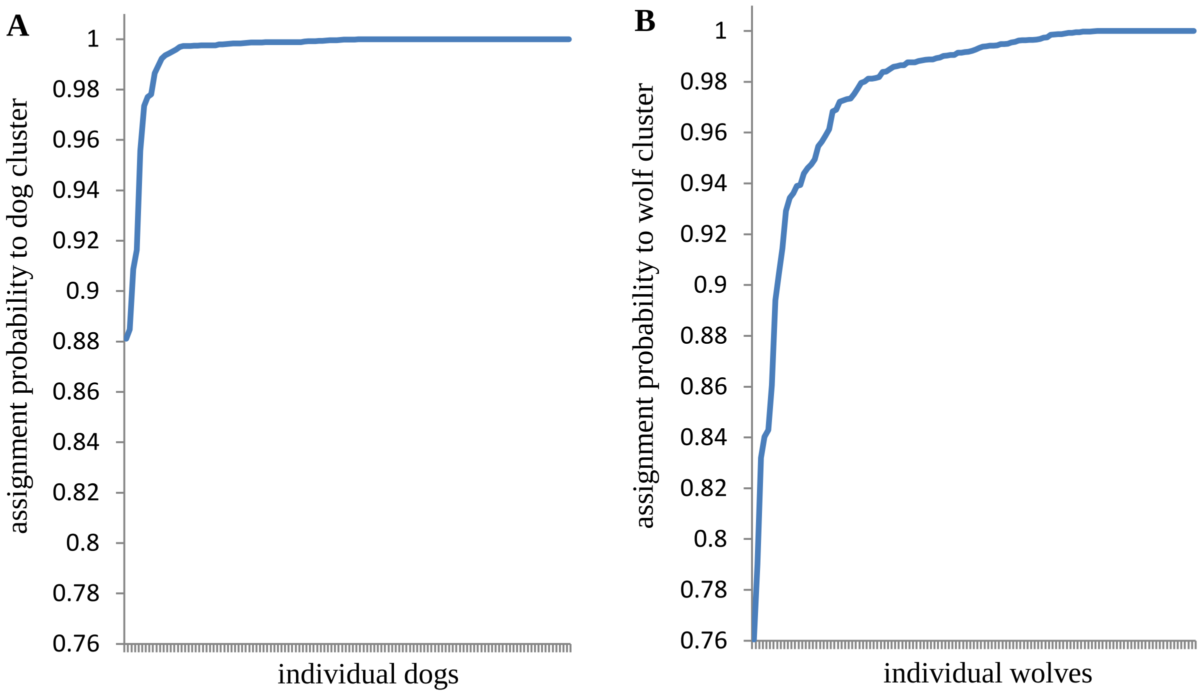

Supporting Figure S5. Comparison of ancestry estimates from LAMP and PCADMIX. (A) Average wolf ancestry proportion estimates from autosomal chromosomes; (B) Number of admixed chromosomes, defined as chromosomes with over 10% of SNP alleles of dog ancestry, as identified in LAMP, or with over 10% of windows of dog ancestry, as identified in PCAdmix. The status of individuals as F2/F3 back-crosses or possible further back-crosses was defined based on combined results of STRUCTURE, ADMIXTURE and LAMP analyses; the PCADMIX analysis was run at a later stage. The outlier, individual #11254, is highlighted on both plots.

A

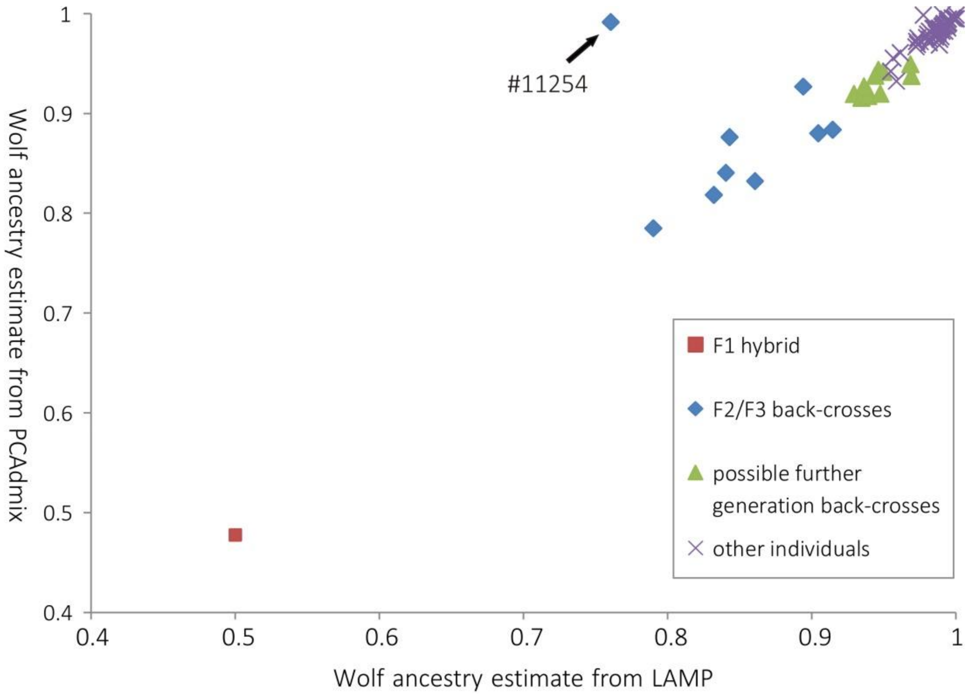

B

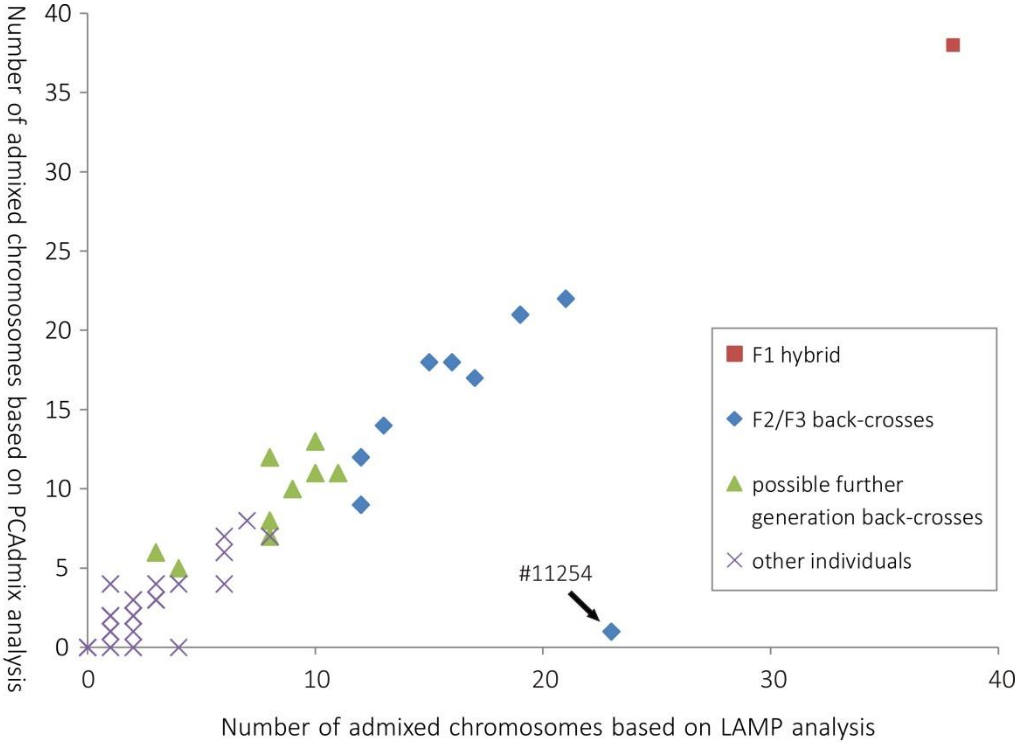

Supporting Figure S6. Evolutionary relationships of X chromosome haplotypes of European wolves and European dog breeds in (A) males and (B) females inferred using the neighbor-joining method. The distances were computed using the p-distance method. Bootstrap support is shown if higher than 50%.

A

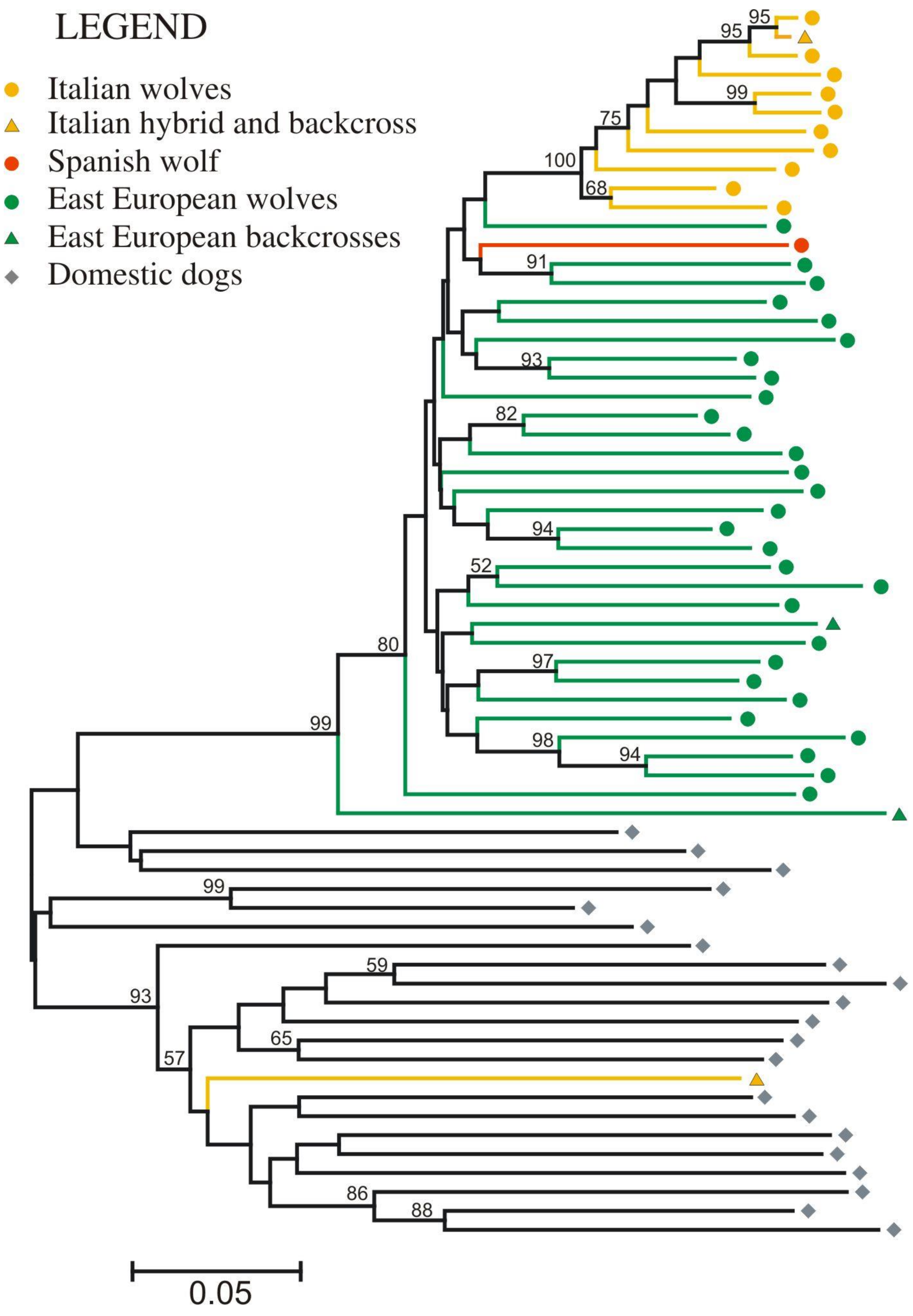

B

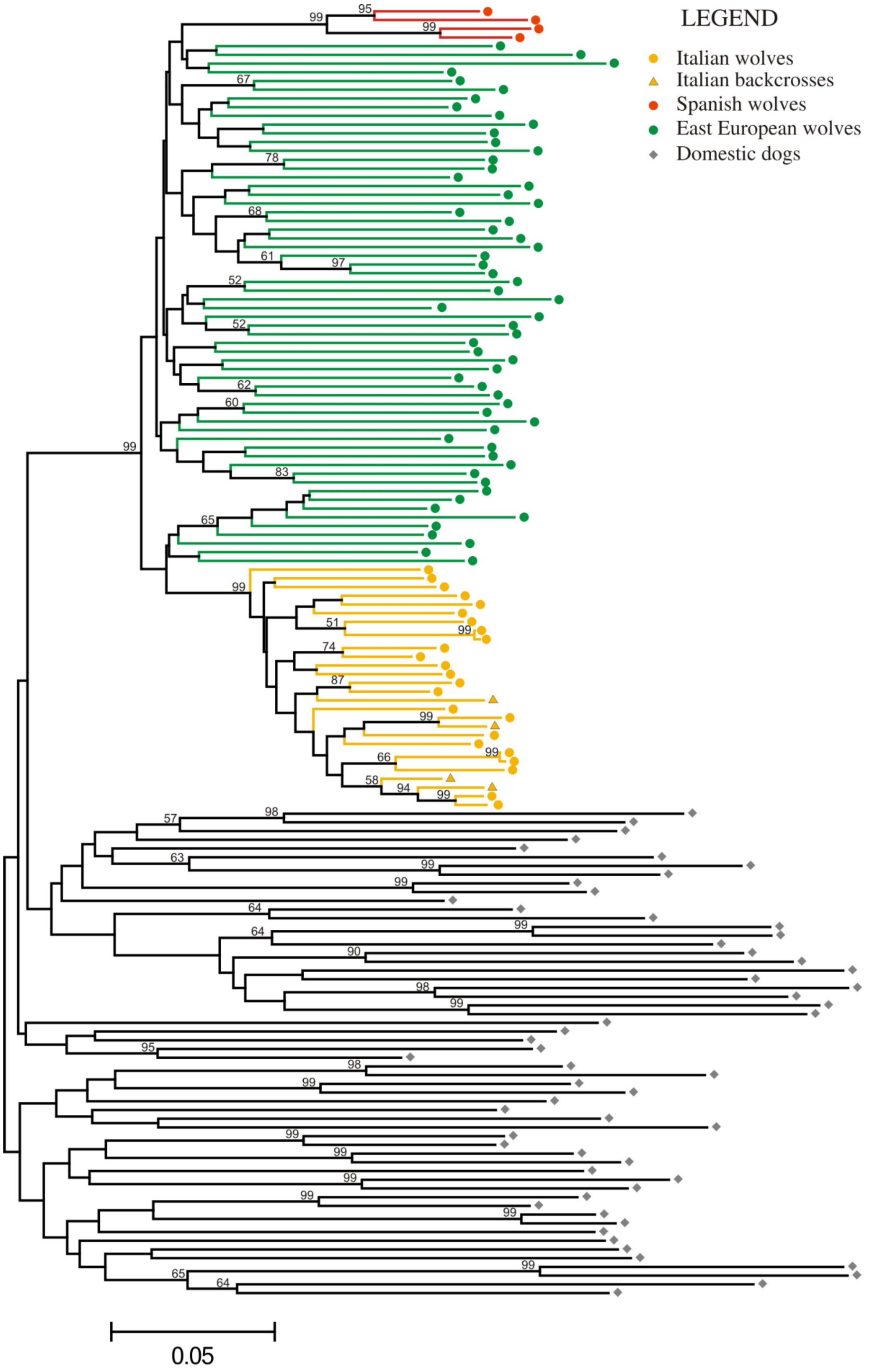
LEGEND
Italian wolves
Italian backcrosses
Spanish wolves
East European wolves
Domestic dogs
0.05
